# Novel TORC1 inhibitor Ecl1 is regulated by phosphorylation in fission yeast

**DOI:** 10.1111/acel.14450

**Published:** 2025-02-05

**Authors:** Hokuto Ohtsuka, Sawa Kawai, Yurika Ito, Yuka Kato, Takafumi Shimasaki, Kazuki Imada, Yoko Otsubo, Akira Yamashita, Emi Mishiro‐Sato, Keiko Kuwata, Hirofumi Aiba

**Affiliations:** ^1^ Department of Basic Medicinal Sciences, Graduate School of Pharmaceutical Sciences, Laboratory of Molecular Microbiology Tokai National Higher Education and Research System, Nagoya University Nagoya Japan; ^2^ Department of Chemistry and Biochemistry Suzuka College, National Institute of Technology (KOSEN) Suzuka Japan; ^3^ Department of Biology, Graduate School of Science Osaka City University Osaka Japan; ^4^ Department of Life Sciences, Graduate School of Arts and Sciences The University of Tokyo Tokyo Japan; ^5^ Life Science Network The University of Tokyo Tokyo Japan; ^6^ Institute of Transformative bio‐Molecules Tokai National Higher Education and Research System, Nagoya University Nagoya Japan

**Keywords:** Ecl1, fission yeas, phosphorylation, starvation, TORC1

## Abstract

Extender of chronological lifespan 1 (Ecl1) inhibits target of rapamycin complex 1 (TORC1) and is necessary for appropriate cellular responses to various stressors, such as starvation, in fission yeast. However, little is known about the effect of posttranslational modifications on Ecl1 regulation. Thus, we investigated the phosphorylation levels of Ecl1 extracted from yeast under conditions of sulfur or metal starvation. Mass spectrometry analysis revealed that Ecl1 was phosphorylated at Thr7, and the level was decreased by starvation. The phosphorylation‐mimetic mutation of Thr7 significantly reduced the effects of Ecl1‐induced cellular responses to starvation, suggesting that Ecl1 function was suppressed by Thr7 phosphorylation. By contrast, regardless of starvation exposure, TORC1 was significantly suppressed, even when Thr7 phosphorylation‐mimetic Ecl1 was overexpressed. This indicated that Ecl1 suppressed TORC1 regardless of Thr7 phosphorylation. We newly identified that Ecl1 physically interacted with TORC1 subunit RAPTOR (Mip1). Based on these evidences, we propose that, Ecl1 has dual functional modes: quantity‐dependent TORC1 inhibition and Thr7 phosphorylation–dependent control of cellular function.

## Graphical abstract

Extender of chronological lifespan 1 (Ecl1), which inhibits the target of rapamycin complex 1 (TORC1), physically interacts with TORC1 subunits and is necessary for appropriate cellular responses to various stressors, such as starvation, in fission yeast. Several functions of Ecl1 are suppressed by Thr7 phosphorylation when nutrients are abundant. We propose that Ecl1 has dual functional modes: quantity‐dependent TORC1 inhibition and Thr7 phosphorylation–dependent regulation of cellular functions.

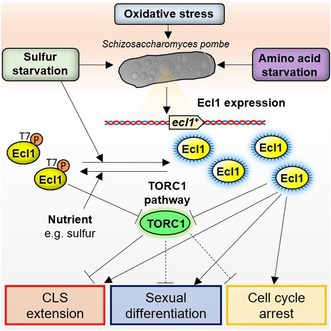

## 1 INTRODUCTION

Research related to aging and lifespan control using various model organisms has revealed that the factors and pathways involved in the response to nutritional starvation are commonly involved in regulating aging and lifespan (Folch et al., 2018; Hansen et al., 2018; Kapahi et al., 2017; Le Couteur et al., 2020; Mirisola & Longo, 2022; Wu et al., 2022). This relationship between nutritional starvation and lifespan regulation is also conserved in unicellular organisms including yeasts. Furthermore, research using budding yeast *Saccharomyces cerevisiae* and fission yeast *Schizosaccharomyces pombe* revealed the involvement of various factors related to nutrition and starvation in pathways that regulate lifespan (Banerjee et al., 2020; Fontana & Partridge, 2015; Ohtsuka, Shimasaki, & Aiba, 2021b; Vega et al., 2022). In yeasts, aging is measured according to its replicative lifespan or chronological lifespan (CLS) (Lin & Austriaco, 2014; Longo et al., 2012). Replicative lifespan refers to the number of daughters a single cell can produce before it dies, and so it is measured as a number of divisions or generations (Lin & Austriaco, 2014; Mortimer & Johnston, 1959; Roux et al., 2010). CLS refers to the duration of survival of nondividing cells and serves as a model for the aging of nondividing cells (e.g., neurons) in higher eukaryotes (Kurauchi et al., 2021; Odoh et al., 2022; Ohtsuka, Shimasaki, & Aiba, 2021c; Ruetenik & Barrientos, 2018). In most yeast CLS experiments, survival rates after entry into the stationary phase are measured (Kurauchi et al., 2017; Lin & Austriaco, 2014; Longo et al., 2012; Matsui et al., 2021; Roux et al., 2010). Over 100 genes, 30 drugs, and approximately 10 nutritional conditions have been found to extend the CLS of *S. pombe* (Batubara et al., 2020; Emami & Ueno, 2021; Fujita et al., 2007; Imai et al., 2020; Martinez‐Miguel et al., 2021; Naito et al., 2014; Ohtsuka et al., 2021d, 2022c; Romila et al., 2021; Su et al., 1996).

Extender of chronological lifespan (Ecl) family proteins are important regulatory factors of CLS (Lin & Austriaco, 2014; Ohtsuka, Shimasaki, & Aiba, 2021c; Roux et al., 2010). Ecl1 was originally identified as a factor that extended the short CLS of a deletion mutant of stress‐responsive mitogen‐activated protein kinase Sty1 (Morigasaki et al., 2013; Sánchez‐Mir et al., 2018; Vivancos et al., 2006), and a similar CLS extension effect of Ecl1 was confirmed in the wild‐type fission yeast cells (Ohtsuka & Aiba, 2017). Three Ecl family genes (*ecl1* ^+^, *ecl2* ^+^, and *ecl3* ^+^) are present in *S. pombe* and one in *S. cerevisiae*, all of which contribute to CLS regulation (Azuma et al., 2012; Ohtsuka & Aiba, 2017). Although *ecl1* ^+^, *ecl2* ^+^, and *ecl3* ^+^ in fission yeast are induced by different signals and mechanisms, their amino acid sequences are similar and each has redundant functions (Brault et al., 2016; Ohtsuka & Aiba, 2017; Ohtsuka et al., 2019, 2021a; Shimasaki et al., 2014). The Zn‐binding protein Ecl1, comprising 80 amino acids, has four Cys residues (Cys6, Cys9, Cys20, and Cys24) that are conserved in Ecl family proteins and considered to bind to Zn (Ohtsuka & Aiba, 2017; Shimasaki et al., 2017). When one of these four Cys residues is replaced with Ser, Ecl function is greatly reduced. Thus, these Cys residues are crucial for Ecl1 function, and the binding of Zn to Ecl1 is considered necessary for proper Ecl1 function (Shimasaki et al., 2017).

Ecl family genes are involved in not only CLS extension but also cellular responses, such as stress tolerance, autophagy, cell cycle arrest, and sexual differentiation, under conditions of starvation of various nutrients, including metals (Ohtsuka et al., 2015, 2023b; Wilson & Bird, 2016). Recently, Ecl family proteins from fission yeast and the insect pathogen *Metarhizium robertsii* were reported as novel factors that suppressed the target of rapamycin (TOR) complex 1 (TORC1), an evolutionarily conserved nutrient‐sensing kinase (Ohtsuka et al., 2022, 2024a). In fission yeast, the TORC1 usually comprises Mip1, Wat1/Pop3, Tco89, Toc1, and Tor2 kinase. It promotes vegetative growth by regulating facultative heterochromatin, protein synthesis, transcription, and ribosome biogenesis while conversely suppressing sexual development and autophagy (Fukuda et al., 2021; Martín & Lopez‐Aviles, 2018; Ohtsuka et al., 2022d; Otsubo et al., 2017, 2018; Shinozaki‐Yabana et al., 2000; Wei et al., 2021). Additionally, TORC1 is involved in the lifespan regulation of various organisms (Folch et al., 2018; Johnson et al., 2013; Kapahi et al., 2017). Reportedly, in fission yeast, the treatment of drugs that inhibit TORC1, such as rapamycin or torin 1, as well as TORC1 inhibition by *tor2* mutations or *tco89* deletion, results in CLS extension (Ohtsuka et al., 2021c, 2024b; Rallis et al., 2013; Rodríguez‐López et al., 2020; Shetty et al., 2019). The intracellular responses caused by Ecl family genes are similar to those caused by TORC1 suppression, suggesting that some functional interactions occur between Ecl family proteins and TORC1, but this relationship remains unelucidated. Recently, it has been reported that a comprehensive analysis suggests a physical interaction between *S. cerevisiae* Ecl1 and Kog1, the fission yeast Mip1 homolog and one of the subunits of TORC1 (Chang et al., 2021).

Although the induction signals and intracellular roles of Ecl1 are gradually being resolved, little is known about its molecular regulation. Protein phosphorylation is a ubiquitous and reversible modification that is critical for regulating multiple intracellular events (Leach & Brown, 2012), and the phosphorylation of Ser61 and Thr63 of Ecl1 has been reported (Halova et al., 2021; Kettenbach et al., 2015; Tay et al., 2019). However, it remains unknown whether other amino acid residues of Ecl1 are also phosphorylated and how this affects Ecl1 function. To this end, we extracted Ecl1 under stress and nonstress conditions and analyzed its phosphorylation levels using mass spectrometry. We further investigated how Ecl1 phosphorylation influenced its function by analyzing substitution mutants of phosphorylated amino acids. Additionally, our investigation into the relationship between Ecl1 and TORC1 revealed that Ecl1 physically interacts with the TORC1 subunit, suggesting that TORC1 suppression by Ecl1 occurs through this physical interaction. Thus, we propose that Ecl1 acts through at least two pathways: a TORC1‐dependent pathway wherein Ecl1 inhibits TORC1 in a dose‐dependent manner and a TORC1‐independent pathway that is regulated by Ecl1 phosphorylation.

## 2 RESULTS

### 2.1 Phosphorylation levels Thr7 and Ser22 in Ecl1 decrease under conditions of sulfur starvation or EDTA stress

**FIGURE 1:**
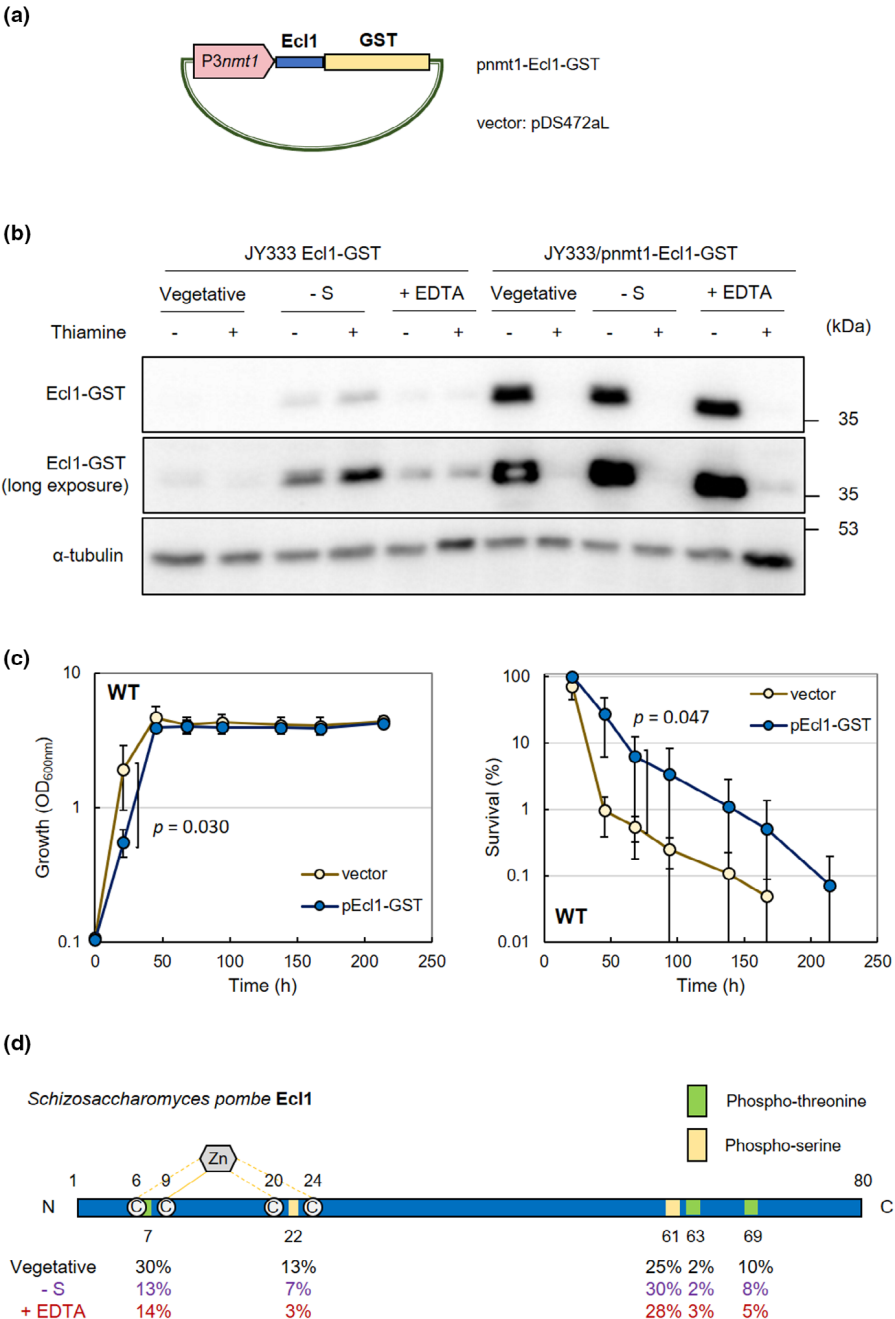
Detection of potential phosphorylation sites of Ecl1‐GST protein. (a) Ecl1‐GST fusion protein was expressed using pnmt1‐Ecl1‐GST, which was prepared by inserting an Ecl1‐GST fragment into vector plasmid pDS472aL. (b) JY333 Ecl1‐GST cells and JY333 cells harboring pnmt1‐Ecl1‐GST were cultured in EMM until OD_600 nm_ = 0.5 and then transferred into EMM with or without 5 mg/L thiamine, 5 mg/L sulfate, and 5 mM EDTA for 3 h. Ecl1‐GST proteins were observed by Western blot analysis. The loading control was α‐tubulin. (c) JY333 cells harboring pDS472aL and pnmt1‐Ecl1‐GST were cultured in synthetic SD medium without thiamine. The growth (left panel) and CLS (right panel) were measured (*n* = 4). (d) The phosphorylation levels of each amino acid residue of the Ecl1‐GST fusion protein under various conditions were measured using mass spectrometry. Sampling conditions are described in Materials and Methods. Phosphorylation of Thr7, Ser22, Ser61, Thr63, and Thr69 was observed. The Cys residues that are considered to bind Zn^2+^ are also specified (Shimasaki et al., 2017).

Ser61 and Thr63 of Ecl1 have been reported as phosphorylated (Halova et al., 2021; Kettenbach et al., 2015; Tay et al., 2019). Furthermore, because of the specificity of its transcriptional expression, Ecl1 is expected to function under stress conditions, such as starvation (Ohtsuka et al., 2023b). First, we aimed to stably purify a large amount of Ecl1 protein from yeast cells to examine changes in Ecl1 phosphorylation during starvation. Therefore, we created plasmid pnmt1‐Ecl1‐GST that expressed a protein with glutathione‐S‐transferase (GST) fused to the C‐terminus of Ecl1 using the P3*nmt1* promoter (Figure 1a). Western blot demonstrated that the expression level of the Ecl1 fusion protein using this plasmid under full expressing condition by thiamine withdrawal was much higher than the amount expressed by the original *ecl1* ^+^ promoter from the chromosome during sulfur starvation (Figure 1b). Furthermore, the overexpression of the Ecl1 fusion protein using this plasmid led to CLS extension, suggesting that the protein was functional (Figure 1c). In addition, Ecl1 fusion protein overexpression slowed the growth rate, similar to a previous report of Ecl1‐overexpression (Ohtsuka et al., 2024a) (Figure 1c). Our above results indicated the successful construction of plasmid pnmt1‐Ecl1‐GST that overexpressed functional Ecl1.

Under nutrient‐rich environments, in which Ecl1 is not induced, its function is supposed to be suppressed. By contrast, under conditions of sulfur or metal starvation, Ecl1 regulates appropriate intracellular responses, such as maintenance of cell viability and sexual differentiation responses (Ohtsuka et al., 2015; Shimasaki et al., 2020). For appropriate functioning of Ecl1, its function may be regulated by not only transcriptional induction but also posttranslational modifications, including phosphorylation. To test this hypothesis, we cultured cells carrying pnmt1‐Ecl1‐GST under three conditions: a nutrient‐rich environment, sulfur starvation, and metal starvation using EDTA. Mass spectrometry analysis of the Ecl1‐GST protein purified from cultures under each condition revealed the phosphorylation of Thr7, Ser22, Ser61, Thr63, and Thr69 of Ecl1 protein in the nutrient‐rich environment (Figure 1d; Figure S1). Under conditions of sulfur or metal starvation, the phosphorylation levels of Ser61 and Thr63 (situated near the C‐terminus) did not change considerably, but those of Thr7 and Ser22 (situated near the N‐terminus) decreased. On the basis of these findings, we decided to focus on Thr7 and Ser22 because their phosphorylation levels decreased under both stress conditions. In this analysis, the overall phosphorylation rate was low (maximum 30%), which might be because the enzyme that phosphorylates Ecl1 was unable to react adequately to the abnormally high levels of Ecl1 protein expressed in cells using the P3*nmt1* promoter (Figure 1b).

### 2.2 Overexpression of phosphomimetic Ecl1 mutations does not effectively restore the Δecls phenotypes

**FIGURE 2:**
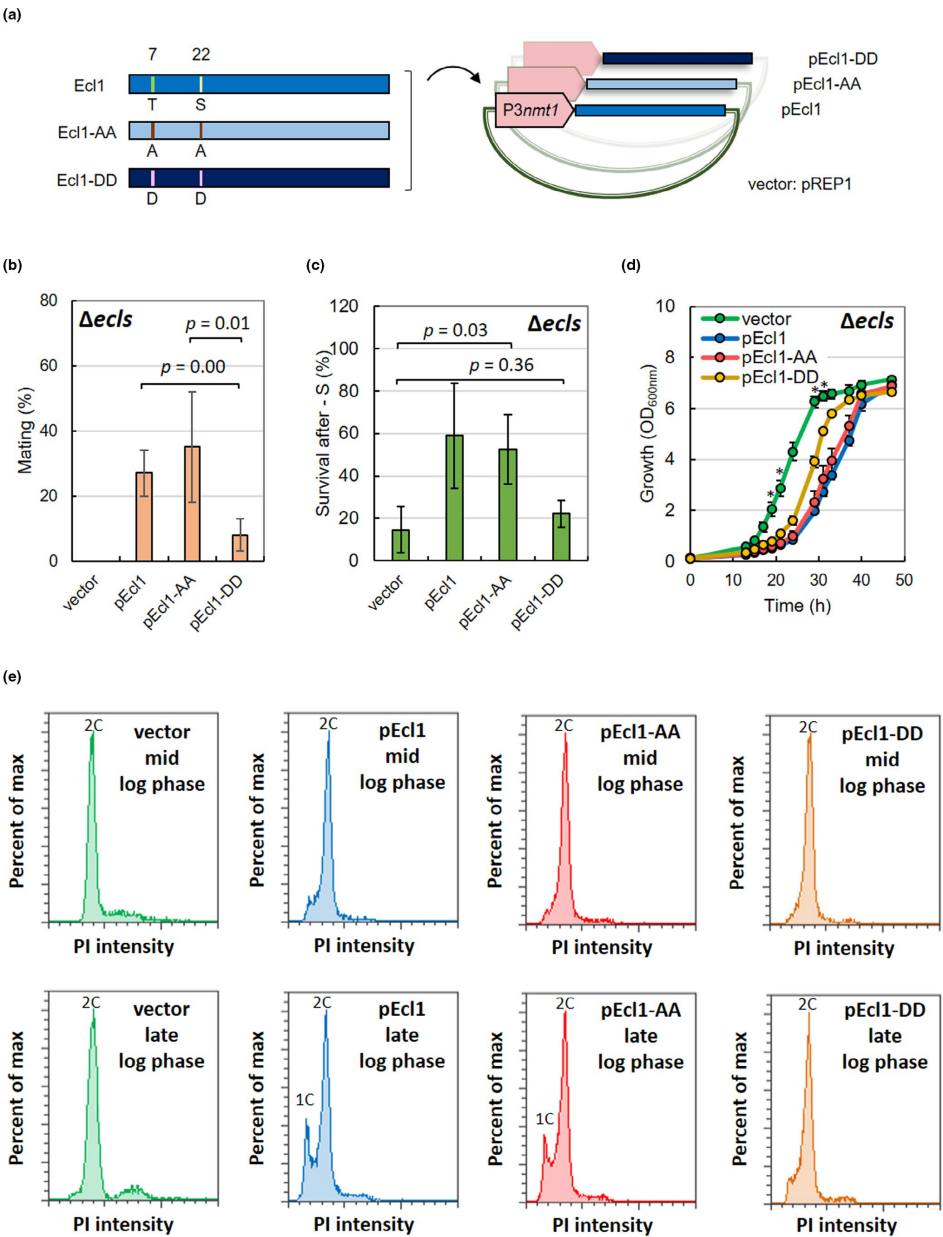
Asp substitutions of Thr7 and Ser22 of Ecl1 suppress Ecl1 activities. (a) Artificial genes encoding Ecl1 in which Thr7 and Ser22 were replaced with Ala (Ecl1‐AA) or Asp (Ecl1‐DD) were created (GeneArt, Thermo Fisher Scientific) and inserted into pREP1 to construct pEcl1‐AA and pEcl1‐DD. (b) The *h* ^90^ homothallic strain JY808Δ*ecl1*Δ*ecl2*Δ*ecl3* harboring pREP1 (vector), pEcl1, pEcl1‐AA, and pEcl1‐DD was cultured in EMM for 3 days, and the mating rates were measured (*n* = 3). (c) JY333Δ*ecl1*Δ*ecl2*Δ*ecl3* cells harboring pREP1 (vector), pEcl1, pEcl1‐AA, and pEcl1‐DD were cultured in SD medium until OD_600 nm_ = 0.5 and then transferred into synthetic SD medium without thiamine and sulfate. The survival rates were measured (*n* = 3) after 3 days of sulfur‐free culture. (d) JY333Δ*ecl1*Δ*ecl2*Δ*ecl3* cells harboring pREP1 (vector), pEcl1, pEcl1‐AA, and pEcl1‐DD were cultured in EMM. Turbidity was chronologically measured to determine the growth rate. Differences in growth rates between vector and pEcl1‐DD and between pEcl1 and pEcl1‐DD were statistically analyzed. *p‐value <0.001. (e) JY333Δ*ecl1*Δ*ecl2*Δ*ecl3* cells harboring pREP1 (vector), pREP1‐Ecl1 (pEcl1), pEcl1‐AA, or pEcl1‐DD were cultured in EMM until OD_600 nm_ = 1.0 (mid‐log phase), and then culture was continued for 6 hours (late‐log phase). DNA content was measured using flow cytometry. The vertical axes of the graphs indicate the maximum percent, and the maximum values of the peak are 100%. All graphs show PI intensities from 0 to 150,000.

Ecl family proteins are necessary for maintaining cell viability during sulfur starvation and for sexual differentiation when cultured to the stationary phase in Edinburgh minimal medium (EMM) or during metal starvation when chelating agents, such as EDTA, are added (Ohtsuka & Aiba, 2017; Ohtsuka et al., 2015; Shimasaki et al., 2020). To analyze how Thr7 and Ser22 phosphorylation affected Ecl1 function, we created plasmids expressing mutant‐Ecl1 proteins in which both of these were changed to Ala (Ecl1‐AA) or Asp (Ecl1‐DD) (Figure 2a; Figure S2). We introduced these plasmids into cells lacking the three *ecl* genes (Δ*ecl1*Δ*ecl2*Δ*ecl3*: Δ*ecls*) and investigated the mating rate during the stationary phase and the survival rate during sulfur starvation to determine whether these plasmids could restore the phenotypes of the Δ*ecls* mutant.

When Δ*ecls* cells were cultured in EMM, mating did not occur, not even in the stationary phase, but this defect was restored by the expression of wild‐type Ecl1 or Ecl1‐AA proteins by plasmids (Figure 2b). This suggested that the Ecl1‐AA mutant protein activity was comparable to that of the wild‐type Ecl1 protein. Meanwhile, when Ecl1‐DD was expressed, the recovery was low, and the degree of recovery was significantly lower than that of Ecl1 and Ecl1‐AA. This suggested that the activity or stability of the Ecl1‐DD mutant protein, which mimics the phosphorylation of Thr7 and Ser22, was reduced compared with the wild‐type Ecl1 protein.

Similar results were observed for survival during sulfur starvation. The survival rate of Δ*ecls* cells harboring empty vectors on day 3 of sulfur starvation was approximately 10%, whereas the survival rates of cells expressing wild‐type Ecl1 or Ecl1‐AA increased markedly, exceeding approximately 50% (Figure 2c). In contrast, no significant increase in survival rate was observed in cells expressing Ecl1‐DD compared with cells harboring empty vectors. These findings supported the possibility that Thr7 and Ser22 phosphorylation suppressed Ecl1 activity.

The overexpression of the *ecl1* ^+^ product slowed the growth rate (Figure 1c). The growth rate of cells with overexpressed Ecl1 that contained each mutation revealed that Ecl1 or Ecl1‐AA overexpression severely retarded growth (Figure 2d). However, although Ecl1‐DD overexpression caused slow growth compared with the vector control, the rate of growth retardation was significantly lower than that of pEcl1 (Figure 2d). Thus, the low inhibitory activity of Ecl1‐DD on the growth rate suggested that Ecl1‐DD activity was reduced.

The mating of fission yeast requires cell cycle arrest in the G_1_ phase (Harigaya & Yamamoto, 2007; Ohtsuka et al., 2022b). Therefore, because mating in the stationary phase is *ecl*‐dependent (Figure 2b), we predicted that G_1_ arrest during the stationary phase also required Ecl1 activity. In Δ*ecls* cells harboring the control vector, cells in the G_1_ phase were not observed during the mid‐log phase or even during the late‐log phase before the stationary phase (Figure 2e). However, in cells harboring pEcl1 or pEcl1‐AA, a population of cells in the G_1_ phase was observed in the late‐log phase (Figure 2e). The number of cells in the G_1_ phase was considerably reduced in cells harboring pEcl1‐DD. These results were consistent with those of the conjugation rate, indicating that *ecl* genes were required for G_1_ arrest before the stationary phase and that phosphorylation of the N‐terminus of Ecl1 played a major role in regulating G_1_ arrest. Furthermore, our findings suggested that the conjugation defect in the stationary phase caused by *ecl* deletion was mainly due to the defect of G_1_ arrest.

### 2.3 Phosphorylation‐mimetic mutation of Thr7 in Ecl1 prevents various cellular responses related to Ecl1

**FIGURE 3:**
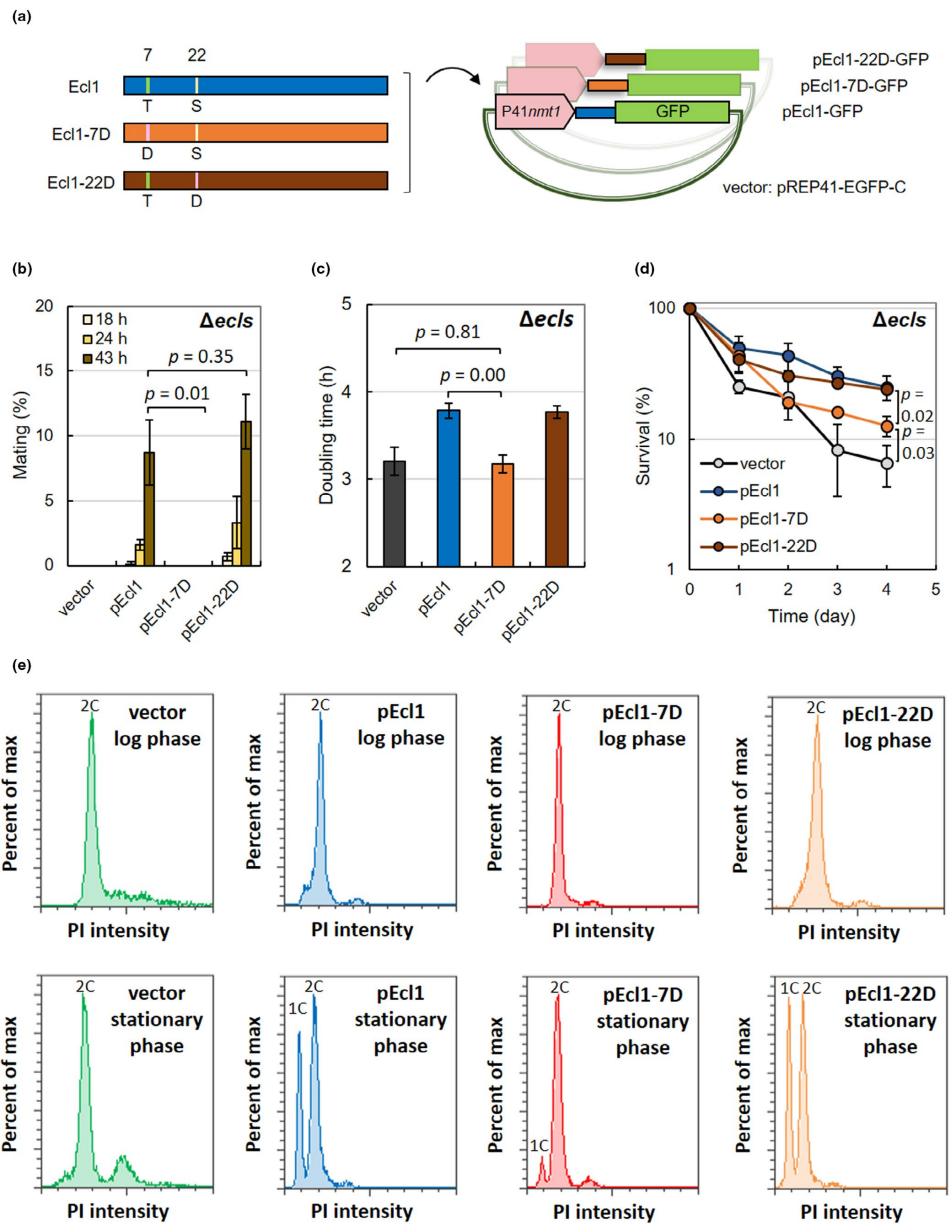
Asp substitution of Ecl1 Thr7 alone is sufficient to suppress its activity. (a) Artificial genes encoding Ecl1 in which Thr7 and Ser22 were replaced with Asp (Ecl1‐7D and Ecl1‐22D, respectively) were created (GeneArt, Thermo Fisher Scientific). These genes were inserted into pREP41‐EGFP‐C to construct pEcl1‐GFP, pEcl1‐7D‐GFP, and pEcl1‐22D‐GFP. (b) The *h* ^90^ homothallic strain JY808Δ*ecl1*Δ*ecl2*Δ*ecl3* harboring pREP41‐EGFP‐C (vector), pEcl1‐GFP (pEcl1), pEcl1‐7D‐GFP (pEcl1‐7D), and pEcl1‐22D‐GFP (pEcl1‐22D) was cultured in EMM, and the mating rates were chronologically measured in the stationary phase (*n* = 3). (c) JY333Δ*ecl1*Δ*ecl2*Δ*ecl3* cells harboring pREP41‐EGFP‐C (vector), pEcl1‐GFP (pEcl1), pEcl1‐7D‐GFP (pEcl1‐7D), and pEcl1‐22D‐GFP (pEcl1‐22D) were cultured in EMM without thiamine, and each doubling time was measured (*n* = 3). (d) JY333Δ*ecl1*Δ*ecl2*Δ*ecl3* cells harboring pREP41‐EGFP‐C (vector), pEcl1‐GFP (pEcl1), pEcl1‐7D‐GFP (pEcl1‐7D), and pEcl1‐22D‐GFP (pEcl1‐22D) were cultured in synthetic SD medium without thiamine until OD_600 nm_ = 0.5 and then transferred into synthetic SD medium without thiamine and sulfate. The survival rates were chronologically measured (*n* = 3). (e) JY333Δ*ecl1*Δ*ecl2*Δ*ecl3* cells harboring pREP1‐EGFP‐C (vector), pEcl1‐GFP (pEcl1), pEcl1‐7D‐GFP (pEcl1‐7D), or pEcl1‐22D‐GFP (pEcl1‐22D) were cultured in EMM until OD_600 nm_ = 0.5, and then culture was continued for 1 day. DNA content was measured using flow cytometry. The vertical axes of the graphs indicate the maximum percent, and the maximum values of the peak are 100%. All graphs show PI intensities from 0 to 200,000.

Ecl1 phosphorylation at Thr7 and Ser22 appeared important for the intracellular responses induced by Ecl1, but it remained unclear whether phosphorylation at both sites was necessary for regulating its activity or whether only one of them was important. To clarify this, we generated phosphomimetic mutants Ecl1‐7D and Ecl1‐22D at either Thr7 or Ser22, respectively (Figure 3a; Figure S2). Moreover, to observe the phenotypic differences in more detail, unlike pEcl1‐AA and pEcl1‐DD, we changed the promoter from P3*nmt1* to P41*nmt1*, which had a lower expression level. We then observed the phenotype of Ecl1 when these mutant Ecl1 proteins were overexpressed.

First, we investigated the conjugation rate in the stationary phase. Overexpression of wild‐type Ecl1 and Ecl1‐22D, but not the empty vector or Ecl1‐7D, rescued the conjugation defect in the stationary phase of Δ*ecls* cells (Figure 3b), suggesting a significant loss of activity in Ecl1‐7D. Furthermore, there was no significant difference in the conjugation rate increased by Ecl1 and Ecl1‐22D overexpression, suggesting that phosphorylation of Ser22 of Ecl1 did not significantly affect Ecl1 activity.

Second, we examined the effect of Ecl1 on growth inhibition. Because Ecl1 overexpression suppressed growth, we investigated the doubling time when Ecl1‐7D and Ecl1‐22D were overexpressed (Figure 3c). The doubling times of the cells harboring the empty vector and pEcl1‐7D were relatively short (both approximately 3.2 h), and there was no significant difference. However, the doubling times of both pEcl1 and pEcl1‐22D were longer (approximately 3.8 h). The doubling time of Ecl1‐7D was significantly shorter than that of wild‐type Ecl1, suggesting that Thr7 phosphorylation significantly reduced Ecl1 activity.

Third, we examined the effects of these Ecl1 mutations on cell survival during sulfur starvation. The survival rates of Δ*ecls* cells harboring pEcl1 or pEcl1‐22D were maintained at relatively high levels during sulfur starvation but were lower in cells harboring the empty vector or pEcl1‐7D (Figure 3d). However, unlike the conjugation rate in the stationary phase, Ecl1‐7D overexpression slightly increased the survival rate compared with the vector control. This suggested that Thr7 phosphorylation in Ecl1 also affected survival under conditions of sulfur starvation. However, because Ecl1‐7D, albeit small, increased viability, the effect of Ecl1 cannot be explained solely by regulation via Thr7 phosphorylation, suggesting additional mechanism that can act even when Thr7 is phosphorylated.

Finally, we investigated cell cycle regulation during entry into the stationary phase. Wild‐type Ecl1 and Ecl1‐22D overexpression caused Δ*ecls* cells in G_1_ arrest in the stationary phase, whereas Ecl1‐7D overexpression was significantly less effective at arresting G_1_ (Figure 3e). This suggested that the phosphorylation of Ser22 of Ecl1 did not significantly affect the phenotype caused by Ecl1. Meanwhile, because phosphomimetic mutations at Thr7 did not restore the G_1_ arrest defect of Δ*ecls* cells, this suggested that Thr7 phosphorylation of Ecl1 suppressed its activity.

These findings indicated that the phosphomimetic mutation of Thr7 caused the loss of various intracellular functions of Ecl1, and it was predicted that fission yeast suppressed Ecl1 activity by phosphorylating Thr7 during nonstress conditions like vegetative growth. In the meantime, we also observed a phenomenon in which Thr7 phosphorylation was insufficient to suppress Ecl1 activity, such as CLS during sulfur starvation.

### 2.4 Phosphorylation mutations of Ecl1 do not affect its protein abundance or subcellular localization

**FIGURE 4:**
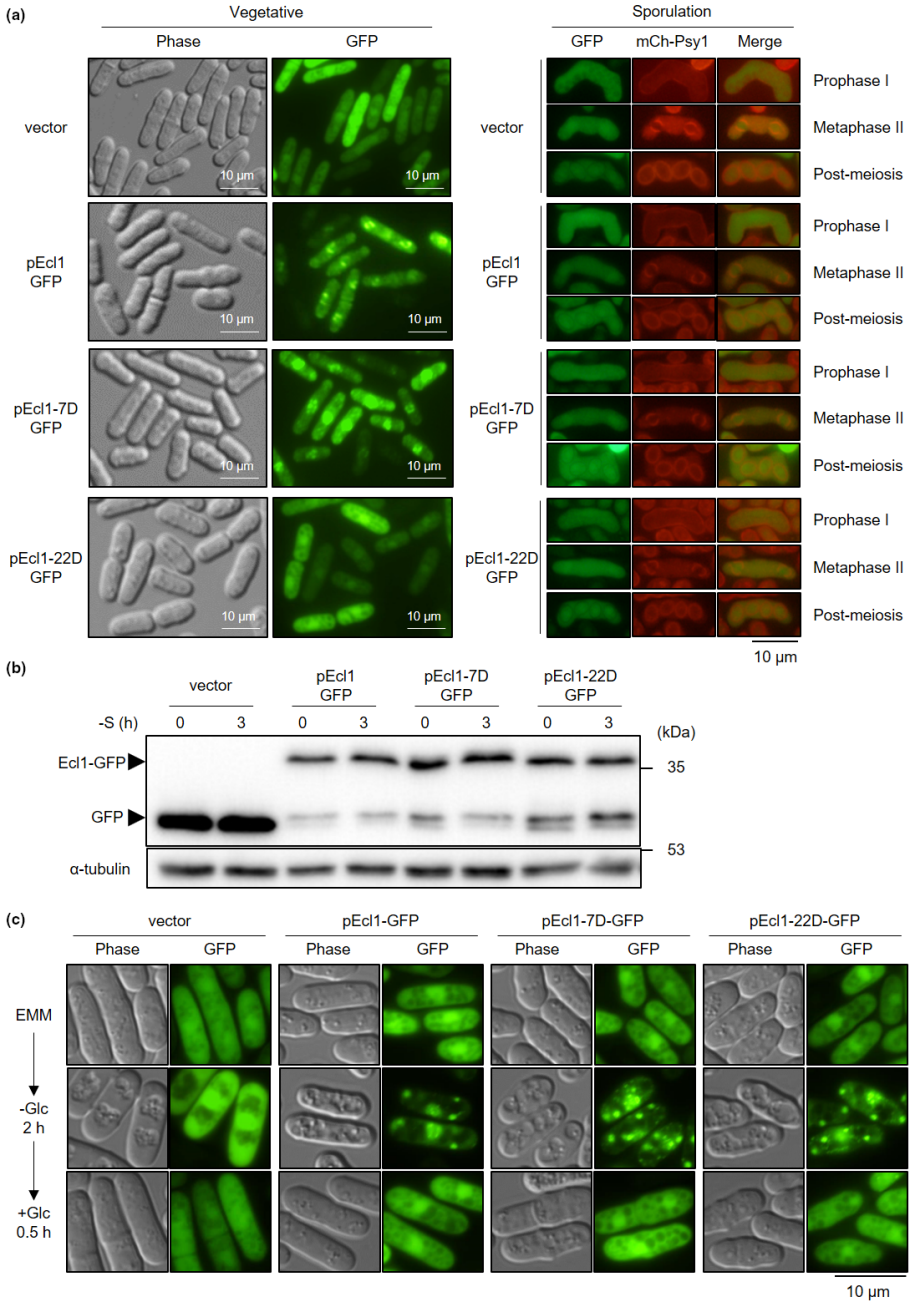
Observation of mutant Ecl1 protein. (a) Cellular localization of Ecl1‐GFP protein at different developmental stages. For the vegetative growth phase (left panels), JY333Δ*ecl1*Δ*ecl2*Δ*ecl3* cells harboring pREP41‐EGFP‐C (vector), pEcl1‐GFP (pEcl1), pEcl1‐7D‐GFP (pEcl1‐7D), or pEcl1‐22D‐GFP (pEcl1‐22D) were cultured in EMM until OD_600 nm_ = 0.5, and then the cells were photographed using a Nikon ECLIPSE Ti microscope. For observation of Ecl1‐GFP during meiosis (right panels), KI53 cells (Imada & Nakamura, 2016) harboring pREP41‐EGFP‐C (vector), pEcl1‐GFP (pEcl1), pEcl1‐7D‐GFP (pEcl1‐7D), or pEcl1‐22D‐GFP (pEcl1‐22D) were sporulated on SPA medium at 28°C for 1 day and photographed using an Olympus BX53 fluorescence microscope with a DP72 digital camera and a cooled charged‐coupled device camera, respectively. Images of GFP (green) and mCherry‐Psy1 (red; used to label the forespore membrane) were taken and overlaid to create merged images using ImageJ/Fiji. (b) Observation of Ecl1‐GFP proteins by Western blot analysis. JY333Δ*ecl1*Δ*ecl2*Δ*ecl3* cells harboring pREP41‐EGFP‐C (vector), pEcl1‐GFP, pEcl1‐7D‐GFP, or pEcl1‐22D‐GFP were cultured in EMM without thiamine until OD_600 nm_ = 0.5 (0 h) and then transferred into EMM without thiamine and sulfate for 3 h. The loading control was α‐tubulin. (c) JY333Δ*ecl1*Δ*ecl2*Δ*ecl3* cells harboring pREP41‐EGFP‐C (vector), pEcl1‐GFP (pEcl1), pEcl1‐7D‐GFP (pEcl1‐7D), or pEcl1‐22D‐GFP (pEcl1‐22D) were cultured in EMM until OD_600 nm_ = 0.5 and then transferred into glucose‐free EMM for 2 h (−Glc). Next, the cells were transferred to EMM containing glucose and cultured for 30 min. Cells were photographed using a Nikon ECLIPSE Ti microscope.

Thr7 phosphorylation in Ecl1 significantly reduced Ecl1 activity. This might be caused by inappropriate cellular localization or reduced protein stability of Ecl1. To investigate this, we observed the intracellular localization of the Ecl1‐GFP fusion protein (Figure 4a). Regardless of mutations at Thr7 and Ser22, the Ecl1‐GFP fusion protein was mainly localized in the nucleus during logarithmic growth and was also observed in the cytoplasm. The Ecl1‐GFP protein did not localize in a specific area at any stage of sporulation, including prophase I, metaphase II, and postmeiosis, and was localized throughout the cell except for the forespore membrane, regardless of the Thr7 or Ser22 mutations. This suggested that phosphorylation of the N‐terminus of Ecl1 was unlikely to significantly affect the intracellular localization of Ecl1.

Next, we investigated the amount of Ecl1‐GFP protein expressed under the P41*nmt1* promoter during logarithmic growth and sulfur starvation using Western blot (Figure 4b). Ecl1‐GFP protein was expressed, even with the Thr7 and Ser22 mutations, and the protein amount did not change considerably regardless of the presence or absence of mutations or changes in extracellular nutritional conditions. Meanwhile, Ecl1‐22D‐GFP produced a relatively large number of degradation products with the same band size as GFP. Although microscopic examination revealed that Ecl1‐22D‐GFP was more highly localized in the cytoplasm compared with wild‐type Ecl1 or Ecl1‐7D, this might have been because the Ecl1‐22D‐GFP degradation products were localized in the cytoplasm, similar to free GFP (Figure 4a).

In addition, we serendipitously found that the localization of Ecl1‐GFP changes based on the extracellular glucose concentration. Upon glucose starvation, Ecl1‐GFP localization changed from its usual nuclear and cytoplasmic localization to multiple dots scattered throughout the cell (Figure 4c). Furthermore, the dot‐like localization returned to the normal nuclear and cytoplasmic localization when cells were transferred to a to a glucose‐containing medium, indicating that Ecl1 is regulated by glucose as well as by amino acids and sulfur, as previously known (Ohtsuka et al., 2023b). Amino acids and sulfur were known to regulate Ecl1 at the transcriptional level; however, extracellular glucose controls the intracellular localization of Ecl1. Therefore, we investigated whether Thr7 mutation affected the altered Ecl1 localization upon glucose starvation (Figure 4c). However, Ecl1‐GFP proteins harboring phosphorylation‐mimetic mutations at Thr7 or Ser22 localized in dot‐like patterns within cells upon glucose starvation—similar to wild‐type Ecl1‐GFP—and reverted to its normal intracellular localization upon glucose restoration, revealing that phosphorylation of the N‐terminus of Ecl1 did not affect the intracellular dot‐like Ecl1 localization.

The above findings demonstrated that although the activity of the mutant Ecl1‐7D protein was expected to be reduced, there was no obvious change in its intracellular localization or protein amount. Therefore, we concluded that the impaired function of Ecl1‐7D was not due to changes in its localization or decreased stability.

### 2.5 Ecl1, which physically interacts with Mip1, reduces TORC1 activity regardless of Thr7 mutation

**FIGURE 5:**
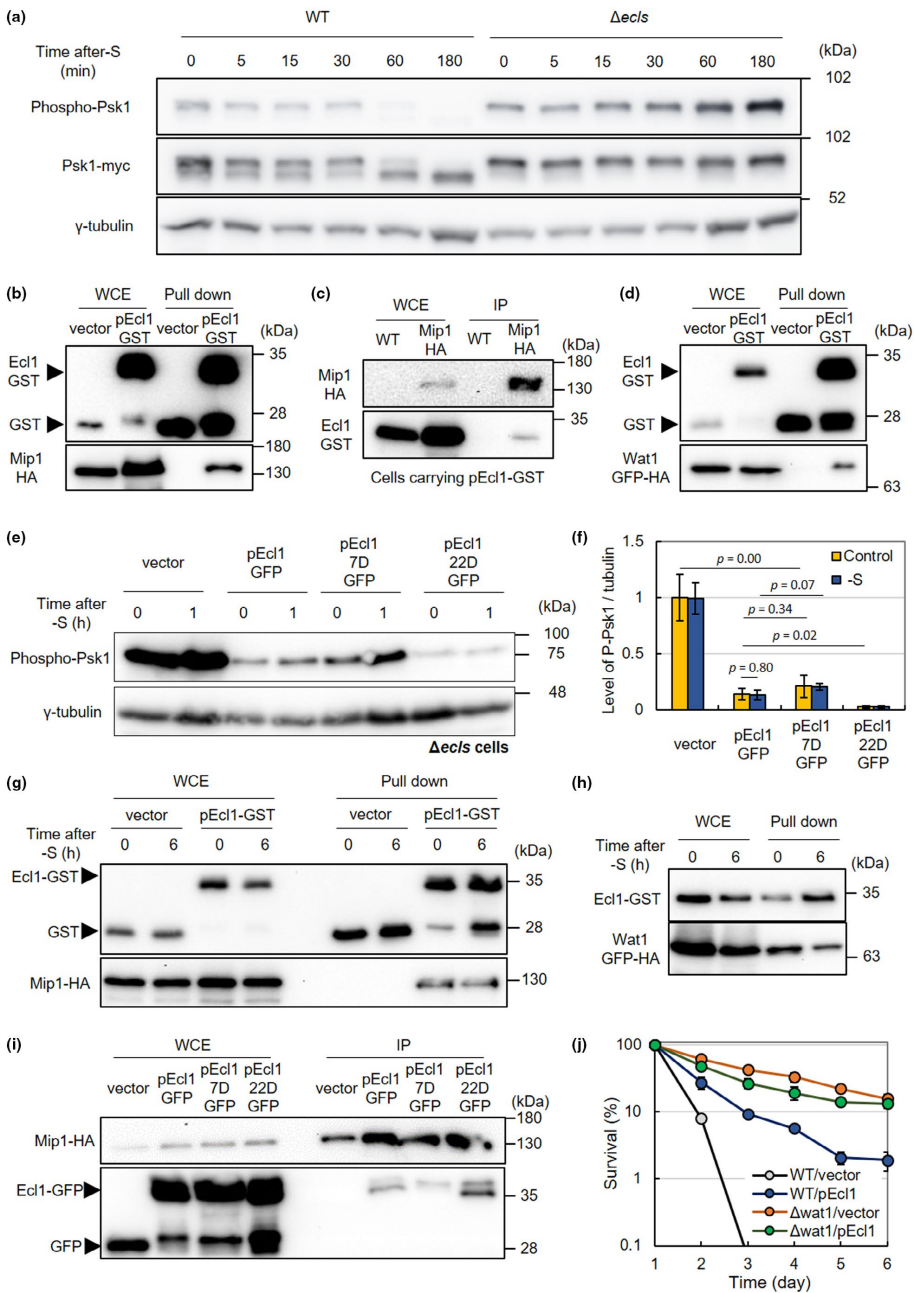
Ecl1 suppresses TORC1 regardless of Thr7 phosphorylation and interacts with TORC1 subunits. (a) JY333 Psk1myc and JY333Δ*ecl1*Δ*ecl2*Δ*ecl3* Psk1myc cells were cultured in EMM until OD_600 nm_ = 0.5 and then transferred into sulfur‐free EMM. Psk1 phosphorylation was observed by Western blot analysis. The loading control was γ‐tubulin. (b) JT176 cells (Matsuo et al., 2007) harboring pDS472aL or pnmt1‐Ecl1‐GST were cultured in EMM until OD_600 nm_ = 0.5 and then transferred into sulfur‐depleted EMM for 6 h. The proteins were purified using COSMOGEL GST‐Accept, and the presence of Ecl1‐GST and Mip1‐HA proteins in whole cell extract (WCE) and pull‐down samples were confirmed by Western blot analysis. (c) JY333 and JT176 cells harboring pnmt1‐Ecl1‐GST were cultured in EMM until OD_600 nm_ = 0.5 and then transferred into sulfur‐depleted EMM for 6 h. The results of immunoprecipitation (IP) with anti‐HA antibodies are shown. (d) FY14831 cells (Hayashi et al., 2009) harboring pDS472aL or pnmt1‐Ecl1‐GST were cultured in EMM until OD_600 nm_ = 0.5 and then transferred into sulfur‐depleted EMM for 6 h. The proteins were purified using COSMOGEL GST‐Accept, and the presence of Ecl1‐GST and Wat1‐GFP‐HA proteins in whole cell extract (WCE) and pull‐down samples were confirmed by Western blot analysis. (e) JY333Δ*ecl1*Δ*ecl2*Δ*ecl3* Psk1myc cells harboring pREP41‐EGFP‐C (vector), pEcl1‐GFP, pEcl1‐7D‐GFP, or pEcl1‐22D‐GFP were cultured in EMM until OD_600 nm_ = 0.5 and then transferred into sulfur‐free EMM for 1 h. Psk1 phosphorylation was observed using Western blot analysis (*n* = 3). (f) The intensity of the phosphorylated Psk1 band of (e) was measured and normalized against the intensity of the γ‐tubulin band. (g) JT176 cells harboring pDS472aL or pnmt1‐Ecl1‐GST were cultured in EMM until OD_600 nm_ = 0.5 (0 h) and then transferred into sulfur‐depleted EMM for 6 h. The result of purification with COSMOGEL GST‐Accept is shown. Ecl1‐GST and Mip1‐HA proteins in whole cell extract (WCE) and pull‐down samples were confirmed by Western blot analysis. (h) FY14831 cells harboring pnmt1‐Ecl1‐GST were cultured in EMM until OD_600 nm_ = 0.5 (0 h) and then transferred into sulfur‐depleted EMM for 6 h. The proteins were purified using COSMOGEL GST‐Accept, and the presence of Ecl1‐GST and Wat1‐GFP‐HA proteins in whole cell extract (WCE) and pull‐down samples were confirmed through Western blot analysis. (i) JT176 cells harboring pREP41‐EGFP‐C (vector), pEcl1‐GFP, pEcl1‐7D‐GFP, or pEcl1‐22D‐GFP were cultured in EMM until OD_600 nm_ = 0.5 and then transferred into sulfur‐free EMM for 6 h. The result of immunoprecipitation (IP) with anti‐HA antibody is shown. (j) JY333 and JY333Δ*wat1* cells harboring pLB‐Dblet (vector) (Ohtsuka et al., 2022a), pEcl1 (Ohtsuka et al., 2022a) were cultured in SD medium. CLS were measured using colony‐forming units (*n* = 3).

TOR is a highly conserved Ser/Thr kinase that regulates various growth‐related functions in response to environmental conditions (Ohtsuka et al., 2023b; Otsubo et al., 2017). The activity of the TORC1 is measured by the phosphorylation level of S6 kinase Psk1, its downstream target (Corral‐Ramos et al., 2022; Nakashima et al., 2012). TORC1 activity was reported to decrease in an *ecl* gene‐dependent manner under conditions of sulfur or phosphate starvation (Ohtsuka et al., 2022a, 2023c) (Figure 5a). Additionally, the overexpression of Ecl1 protein or its homolog has been reported to suppress TORC1 (Ohtsuka et al., 2024a). These findings indicated that Ecl proteins were potent TORC1 inhibitors, although the exact mechanism remains unelucidated.

Meanwhile, the cellular responses to sulfur starvation, including autophagy, are relatively slow (Corral‐Ramos et al., 2022; Shimasaki et al., 2020). We investigated how long it takes for sulfur starvation to suppress TORC1. Therefore, the phosphorylation level of Psk1 began to decrease in an Ecl protein–dependent manner approximately 1 h after sulfur starvation (Figure 5a), indicating that it takes approximately 1 h for the sulfur starvation signal to be transmitted to TORC1.

Chang et al., used biomolecular fluorescence complementation screening to identify 130 proteins, including budding yeast Ecl1, as potential interactors with Kog1, a subunit RAPTOR of TORC1 in budding yeast (Chang et al., 2021). This suggested that *S. pombe* Ecl1 interacts with RAPTOR of TORC1 to suppress TORC1. To investigate this possibility, we introduced pnmt1‐Ecl1‐GST into fission yeast cells with HA‐tagged RAPTOR (Mip1) and examined the interaction by GST pull‐down assay (Figure 5b). The pulled‐down Ecl1‐GST protein was collected along with the Mip1‐HA protein, suggesting a physical interaction between Ecl1 and Mip1. Additionally, immunoprecipitation with HA antibody resulted in the coprecipitation of Ecl1‐GST with Mip1‐HA (Figure 5c). This supported the physical interaction of Ecl1 with Mip1 and suggested that Ecl1‐mediated repression of TORC1 might be regulated by the physical interaction between Ecl1 and Mip1. Furthermore, the pulled‐down Ecl1‐GST protein was also collected along with the Wat1‐GFP‐HA protein, suggesting a physical interaction between Ecl1 and Wat1 (Figure 5d). Wat1 is a mammalian Lst8 ortholog and another TORC1 subunit (Panigrahi et al., 2023).

Our results thus far demonstrated that various Ecl1 functions were suppressed by N‐terminus phosphorylation. Therefore, we investigated whether N‐terminus phosphorylation of Ecl1 was involved in reducing TORC1 activity by measuring the phosphorylation level of Psk1 in Δ*ecls* cells expressing Ecl1 with a mutation at the phosphorylation sites (Figure 5e,f). Although Ecl1‐7D protein had little Ecl1 function as far as various Ecl‐dependent phenotypes were concerned (Figure 3), wild‐type Ecl1, Ecl1‐22D, and Ecl1‐7D significantly reduced TORC1 activity. Furthermore, the Ecl1‐overexpression reduced TORC1 activity regardless of the presence or absence of sulfur (Figure 5e,f). In other words, even during the logarithmic growth phase, when Thr7 is phosphorylated and Ecl function is suppressed, Ecl1 overexpression sufficiently reduces TORC1 activity. These results indicated that phosphorylation of the N‐terminus of Ecl1 had little effect on TORC1 repression and that the expression of Ecl1, rather than starvation conditions, was important for TORC1 repression.

If the level of Ecl1 expression was important for TORC1 repression, we predicted that binding of Ecl1 to Mip1 would occur with or without sulfur starvation in cells Ecl1 protein was overexpressed. We verified this and confirmed that Ecl1 interacts with Mip1 and Wat1 under logarithmic growth phase and sulfur starvation condition (Figure 5g,h). This supports the idea that expression of Ecl1 itself is more important for binding to TORC1 subunits and TORC1 suppression than the extracellular environment, such as sulfur starvation.

We investigated the effect of phosphorylation mutations of Ecl1 on the binding of TORC1 subunit Mip1 to Ecl1 (Figure 5i). Mip1‐HA protein did not coprecipitate with GFP but did coprecipitate wild‐type Ecl1 and with Ecl1 proteins with mutations at Thr7 or Ser22. These results supported the binding of Ecl1 to TORC1 and consequent inhibition of TORC1, even when Ecl1 was phosphorylated at Thr7. Based on the above, we concluded that Ecl1 had some intracellular functions that were dependent on Thr7 phosphorylation and others that depended on binding to Mip1 and on TORC1 inhibition, independent of Thr7 phosphorylation.

Finally, we investigated the effects of Ecl1 and TORC1 on lifespan. Although TORC1 subunits—Tor2 and Mip1—are essential, Wat1 is a nonessential subunit (Panigrahi et al., 2023). If CLS extension by Ecl1 is mediated by the same pathway as TORC1, no additive CLS extension would be observed if Ecl1 was overexpressed in a TORC1 subunit–deficient cell. Then, we made a deletion mutant of Wat1, a TORC1 subunit confirmed to interact with Ecl1, and investigated CLS of Δ*wat1* cells when *ecl1* ^+^ was overexpressed (Figure 5j). Although *wat1* ^+^ deletion or *ecl1* ^+^ overexpression extended the CLS, overexpression of *ecl1* ^+^ in the *wat1*‐deficient cells did not result in further CLS extension, suggesting that Ecl1 and Wat1 function in CLS extension through the same pathway. However, because Wat1 is a component of TORC1 and TORC2 (Ahamad et al., 2018), further experiments are required to clarify whether Ecl1‐induced CLS extension is exclusively mediated by TORC1.

### 2.6 AlphaFold predicts Ecl1 structure

**FIGURE 6:**
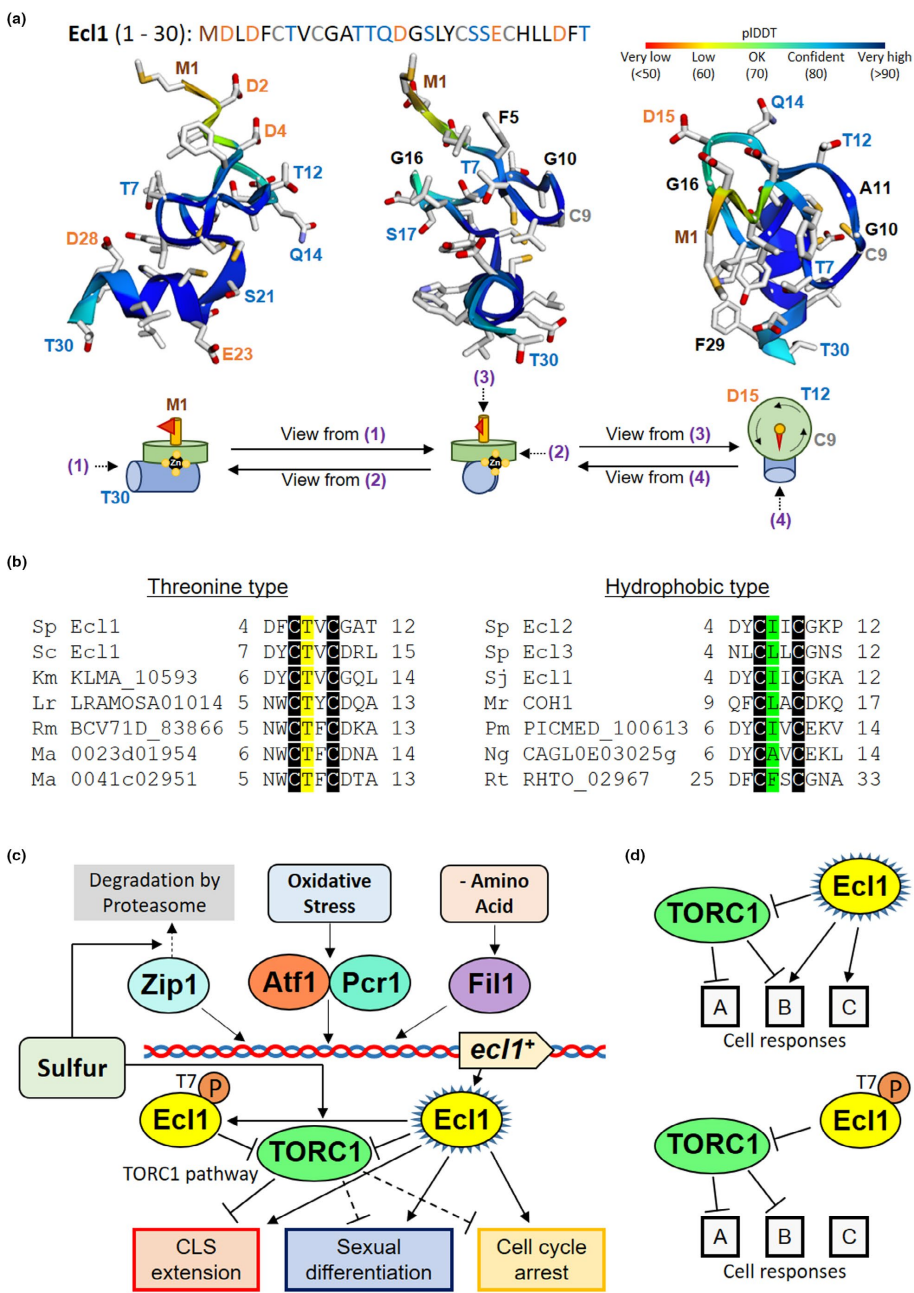
Model of the intracellular function of Thr7 phosphorylation of Ecl1. (a) Structure of the N‐terminus (from Met1 to Thr30) of Ecl1 from three different perspectives as predicted using AlphaFold (pLDDT = 85.2). The lower section presents simple illustrations of the N‐terminal structures of Ecl1 showing the perspective from which the structure is viewed. Four Cys residues (Cys6, Cys9, Cys20, and Cys24) grouped at the center of the N‐terminal structure of Ecl1 are considered to bind to Zn. (b) Thr7 of Ecl1 is conserved in several Ecl homologs, including *S. cerevisiae* Ecl1. However, several other Ecl homologs, including *S. pombe* Ecl2, have a hydrophobic amino acid in place of Thr. The same Thr type as *S. pombe* Ecl1 was found in *S. cerevisiae* Ecl1, *Kluyveromyces marxianus* KLMA_10593, *Lichtheimia ramosa* LRAMOSA01014, *Rhizopus microsporus* BCV71DRAFT_83866, *Mucor ambiguus* MAM1_0023d01954, and *M. ambiguus* MAM1_0041c02951. The same hydrophobic amino acid type as *S. pombe* Ecl2 was found in *S. pombe* Ecl3, *Schizosaccharomyces japonicus* Ecl1, *M. robertsii* COH1, *Pichia membranifaciens* PICMEDRAFT_100613, *Nakaseomyces glabratus* CAGL0E03025g, and *Rhodotorula toruloides* RHTO_02967. (c) Phosphorylation of Thr7 of Ecl1 suppresses some intracellular functions of Ecl1, including CLS extension, sexual differentiation, and cell cycle arrest. However, TORC1 activity is suppressed by Ecl1 overexpression regardless of Thr7 phosphorylation. The transcriptional expression of *ecl1* ^+^ is induced by amino acid starvation and oxidative stress. Sulfur starvation not only induces *ecl1* ^+^ transcription via the transcription factor Zip1 but also leads Thr7 dephosphorylation of Ecl1. (d) Ecl1 is considered to mediate cellular responses through two pathways: TORC1‐dependent and ‐independent responses. For example, Psk1 phosphorylation is suppressed regardless of the Thr7 phosphorylation of Ecl1 (Figure 5e), and thus, it is an example of mainly TORC1‐dependent “Cellular response A.” Because CLS under sulfur starvation was partially upregulated by Ecl1‐7D overexpression (Figure 3d), it is an example of “Cellular response B" that is partially dependent on TORC1. Ecl1 phosphorylated at Thr7 inhibits TORC1 but abolishes mating during stationary phase (Figure 3b), and thus, an example of a TORC1‐independent “Cellular response C” is the mating response.

Predictions of protein structure using the artificial intelligence program AlphaFold can be directly used as structural models or indirectly used as an aid to experimental structure determination using X‐ray crystallography or nuclear magnetic resonance spectroscopy (Laurents, 2022; Marcu et al., 2022; Mirdita et al., 2022). The N‐terminal 1–30 region of Ecl1 was predicted by AlphaFold with relatively high confidence, with a predicted local distance difference test (pLDDT) value of 85.2 (Figure 6a). According to this prediction, the N‐terminal structure of Ecl1 consisted of a head portion from Met1 to Phe5, a planar single‐turn portion from Cys6 to Tyr19, and a portion that projected from Cys20 in the opposite direction to Met1 to form an α‐helix including Cys24. The four cysteines (Cys6, Cys9, Cys20, and Cys24) were clustered at one site, forming a Zn finger‐like structure without a finger, with Cys6 and Cys9 positioned in opposite directions on the plane, Cys20 located near the start of the α‐helix, and Cys24 located on the α‐helix.

### 2.7 Conservation of two types of Ecl proteins: A Thr type and a hydrophobic amino acid type

Our study provided data showing that Ecl1 phosphorylation at Thr7 is responsible, in part, for Ecl1 function regulation and the induction of appropriate cellular responses. However, it was unknown whether this phosphorylatable Thr7 was conserved in other Ecl family proteins. A search of several other Ecl proteins and factors containing the Ecl family domain revealed conservation of the Thr7 moiety of Ecl1 in some members, but it was often replaced by hydrophobic amino acids in other members (Figure 6b). A representative example of the Thr type, like *S. pombe* Ecl1, is *S. cerevisiae* Ecl1, and representative examples of the hydrophobic amino acid type are *S. pombe* Ecl2 and Ecl3. Additionally, the COH1 protein of *M. robertsii* was recently reported to have a similar function to the Ecl proteins of *S. pombe* and also had a hydrophobic amino acid type (Ohtsuka et al., 2024a). In our study, because Ecl1‐AA had the same function as wild‐type Ecl1 (Figure 2), the hydrophobic amino acid type may have sufficient Ecl1 function regardless of stress, such as starvation. Furthermore, the activity of Ecl proteins that contain the conserved Thr residue may be regulated via phosphorylation in other organisms, including budding yeast.

## 3 DISCUSSION

Ecl1 is a Zn‐binding protein, and Zn is considered to be retained by four cysteine residues of Ecl1: Cys6, Cys9, Cys20, and Cys24 (Shimasaki et al., 2017). However, the three‐dimensional structure of Ecl1 remains undetermined. Thr7 of Ecl1 is located close to these four Cys residues (Figure 1d) and is the site of phosphorylation affecting Ecl1 function. The binding of Zn in Zn finger proteins is thought to contribute to stabilize the three‐dimensional structure of the protein (Frankel et al., 1987; Low et al., 2002; Párraga et al., 1988). Thus, the binding of Ecl1 to Zn may enhance the structural stability of the N‐terminus. The CLS extension activity of Ecl1 was reported to be abolished by all Ser‐substitution mutants of Ecl1 for each Cys residue (Shimasaki et al., 2017). Hence, the destruction of these Cys structures via Thr7 phosphorylation could disrupt Ecl1 function. However, the protein structure predicted by AlphaFold shows Thr7 close to these four Cys residues (Figure 6a), with the side chain of Thr7 positioned in the opposite direction. Therefore, Thr7 phosphorylation may not directly interfere with the binding of Zn to the four Cys residues.

It is assumed that the suppression of Ecl1 function by Thr7 phosphorylation primarily occurs under nutrient‐rich conditions (Figure 1d). Several kinases are potential candidates for phosphorylation of Ecl1 at Thr7. Major Ser/Thr kinases that are relatively highly active under nutrient‐rich conditions in fission yeast include cAMP‐dependent protein kinase (PKA), which senses nutritional status and responds to the associated intracellular response; Pat1, an inhibitor of sexual differentiation response; Cdc2, a cyclin‐dependent kinase (CDK) cell cycle regulator; and TOR kinases (Corral‐Ramos et al., 2022; García‐Blanco & Moreno, 2019; Laboucarié et al., 2017; Nishida et al., 2019; Otsubo & Yamamoto, 2012; Toyoda & Saitoh, 2021; Yamashita et al., 2017; Yanagida, 2009). PKA phosphorylates the RRXS motif, including Thr253 of Rst2, a PKA target (Castañeda‐Bueno et al., 2017; Higuchi et al., 2002), but Ecl1 does not contain this motif. Pat1 phosphorylates Ser218 and Thr173 of Ste11 (Li & McLeod, 1996) and Ser438 and Thr527 of Mei2 (Watanabe et al., 1997), which have Arg at the −3 position and Ser/Thr at the −2 position, but not Thr7 of Ecl1. The consensus phosphorylation site for Cdc2 is (Ser/Thr)Pro (Rachfall et al., 2014). For example, Cdc2 has been confirmed to phosphorylate Thr314, Ser462, and Ser481 of Fkh2 (Shimada et al., 2008; Swaffer et al., 2016), Ser248, Ser326, Thr429, Ser499, Thr502, and Ser533 of the septation initiation network inhibitor Byr4 (Rachfall et al., 2014; Swaffer et al., 2016), and Ser3, Ser66, Ser84, Thr89, Thr104, Ser113, Ser118, Ser143, Ser332, Ser334, Thr351, and Thr379 of the CDK phosphatase Cdc25 (Lu et al., 2012; Swaffer et al., 2016). However, the N‐terminus of Ecl1 does not have these sites. Studies on mammalian TOR have reported that its consensus motif prefers Pro, hydrophobic residues Leu and Val, and aromatic residues Phe, Trp, and Tyr at the +1 position (Hsu et al., 2011). The known target sites of TOR kinases in fission yeast fit this consensus motif, including targets of TORC1, namely Thr415 of Psk1 (Nakashima et al., 2012), Ser35, Ser39, and Ser343 of Mei2 (Otsubo et al., 2014), and Thr82 and Thr88 of Ste11 (Otsubo et al., 2017), and targets of TORC2, namely Ser527 and Ser546 of Gad8 (Cohen et al., 2014). This also applies to *S. pombe* Ecl1, *S. cerevisiae* Ecl1, and *Kluyveromyces marxianus* Ecl1 homolog, which have Val at the +1 position, and other Thr type Ecl1 homologs that also have aromatic residues at the +1 position (Figure 6b). Although Ecl1 suppresses TORC1 activity, TORC1 itself may also inhibit Ecl1 by phosphorylating Ecl1 at Thr7 under nonstress conditions. Further analysis of the Ecl protein and TOR complex at the molecular level is necessary to provide new insights into TOR kinase regulation.

In contrast to the pronounced effect of Thr7 phosphorylation, the effect of Ser22 in Ecl1 was minor. Notably, Ecl1‐22D, a phosphorylation mimetic of Ser22, showed slightly stronger phenotypes than wild‐type Ecl1 in some cases. For example, in cells harboring pEcl1‐22D‐GFP, the number of cells entering G1 phase after stationary phase was slightly higher than that of pEcl1‐GFP (Figure 3e) and the inhibitory effect of TORC1 was stronger than that of pEcl1‐GFP (Figure 5e,f). Furthermore, Ecl1‐22D‐GFP seemed localized in the cytoplasm, in which Mip1 is localized (Morozumi et al., 2021), rather than in the nucleus (Figure 4a). Although a reason for the cytoplasmic localization of Ecl1‐22D‐GFP is considered to be its instability (Figure 4b), immunoprecipitation of Mip1‐HA coprecipitated more Ecl1‐22D‐GFP than wild‐type Ecl1‐GFP (Figure 5i), suggesting that phosphorylation of Ser22 might promote the cytoplasmic localization of Ecl1 and enhance its interaction with the TORC1 subunit, thereby enhancing its function. In fact, Ser22 is not conserved in other fungal Ecl homologs and is mostly conserved as a negatively charged amino acid that mimics phosphorylation (Figure S3).

Herein, the deletion of *wat1* ^+^ extended CLS of *S. pombe* when cultured in synthetic dextrose (SD) medium (Figure 5j). Meanwhile, CLS is reportedly reduced during nitrogen starvation owing to the *wat1* deletion (Zahedi et al., 2020). This may be because Wat1 is a component of TORC1 and TORC2 and is involved in their regulation (Ahamad et al., 2018; Matsuo et al., 2007). Deletion of Tor1, the kinase of TORC2, extends CLS in SD medium but decreases it in YE, suggesting that the effect of TORC2 on CLS depends on the growth medium (Ohtsuka, Shimasaki, & Aiba, 2021b; Rallis et al., 2013). In addition, the deletion of Gad8, a downstream factor of TORC2 (Cohen et al., 2014), shortened the CLS under nitrogen starvation (Zahedi et al., 2020), suggesting that TORC2 pathway inhibition leads to a short CLS under nitrogen starvation. Because our CLS assay of Δ*wat1* cells was performed in SD medium, in which TORC2 suppression extends CLS, the loss of Wat1 may have contributed to CLS extension.

Transcriptional regulation via specific transcription factors in response to various stress conditions, including starvation, is considered as the main regulation of Ecl1 function (Ohtsuka et al., 2023b) (Figure 6c). However, our study demonstrates that the regulation of Ecl1 function was mediated not only at the transcriptional level but also through posttranslational modification via Thr7 phosphorylation. Ecl1 regulation by Thr7 phosphorylation suppresses certain functions of Ecl1, such as cell cycle control and sexual differentiation in the stationary phase, growth rate, and survival during starvation (Figure 3), while minimally affecting the suppression of the Ecl1 target TORC1 (Figure 5). Therefore, we suggest that dephosphorylation of Thr7 of Ecl1 is necessary for these Ecl1‐induced cellular responses during starvation. Meanwhile, the repression of TORC activity by Ecl1 appears to depend on its abundance regardless of nutrient availability in the environment (Ohtsuka et al., 2022a, 2023c, 2024a) (Figure 5e). Thus, Ecl1 functions through two mechanisms dependent on the quality of Thr7 phosphorylation and the quantity of the protein. Simultaneously, Ecl1‐7D considerably suppresses TORC1 but does not rescue some phenotypes. This means that while many phenotypes associated with Ecl1 are similar to those regulated by TORC1 suppression, not all Ecl1 functions are mediated by TORC1 suppression. Thus, despite Ecl1 repressing TORC1, it is suggested that Ecl1 also targets other pathways and mediates cellular responses independent of TORC1 signaling (Figure 6d). Furthermore, based on studies on *S. cerevisiae* and *M. robertsii* (Chang et al., 2021; Ohtsuka et al., 2024a), the physical interaction between Ecl1 and TORC1 subunits and the TORC1‐suppressing effect of Ecl1 are conserved across species. Thus, the Ecl protein likely plays a role in the evolutionarily conserved functions of physical interaction with and suppression of TORC1, at least in fungi. Although TORC1 is highly conserved in eukaryotes, Ecl proteins appear to be conserved only in fungi. This suggests that further studies focusing on fungal Ecl proteins and TORC1 could reveal fungus‐specific regulation of TORC1.

## 4 MATERIALS AND METHODS

### 4.1 Strains and growth media

*S. pombe* strains are listed in Table S1. The yeast strains were cultured in SD medium or EMM supplemented with 40 μg/mL adenine (Moreno et al., 1991). For sulfur starvation, sulfur‐free SD medium (Shimasaki et al., 2020) was used. Meiosis was observed using sporulation agar (SPA) medium (1% glucose, 0.1% KH_2_PO_4_, 0.0001% calcium pantothenate, 0.0001% nicotinic acid, 0.001% inositol, 0.000001% biotin, and 1.5% agar) (Munz & Leupold, 1970). Cells were cultured at 30°C unless otherwise stated.

### 4.2 Construction of JY333 Ecl1‐GST and JY333Δwat1

We fused GST to the C‐terminus of the Ecl1 protein on the chromosome of JY333 as previously described (Kurauchi et al., 2021). The primers used are listed in Table S1. JY333Δ*wat1* strain was constructed by crossing Δ*wat1* (Δ*pop3*) mutant provided by Bioneer (http://us.bioneer.com/ products/spombe/spombeoverview.aspx) with JY333 (Maruyama et al., 2022).

### 4.3 Construction of the pnmt1‐Ecl1‐GST plasmid

We constructed pnmt1‐Ecl1‐GST by inserting the *ecl1* ^+^ open reading frame into the *Bgl*II/*Not*I sites at the N‐terminus of GST in pDS472aL (Ma et al., 2016; Siam et al., 2004). The primers are listed in Table S1.

### 4.4 Construction of the plasmids harboring mutant ecl1^+^

To generate pEcl1‐DD and pEcl1‐AA, the DNA fragments of each mutant *ecl1* ^+^ were inserted into the *Nde*II/*Bam*HI sites in pREP1 (Maundrell, 1993). To generate pEcl1‐GFP, pEcl1‐7D‐GFP, and pEcl1‐22D‐GFP, the DNA fragments of each mutant *ecl1* ^+^ were inserted into the *Nde*I/*Bam*HI sites in pREP41‐EGFP‐C (Craven et al., 1998). The DNA fragments of each mutant *ecl1* ^+^ were artificially synthesized by Thermo Fisher Scientific via its GeneArt gene synthesis service. The primers are listed in Table S1.

### 4.5 Purification of the Ecl1‐GST protein for analysis of phosphorylated amino acid residues

JY333/pnmt1‐Ecl1‐GST cells were cultured in EMM to an optical density of 0.5 at 600 nm (vegetative sample) and then transferred into sulfur‐free EMM (Shimasaki et al., 2020) for 8 h (−S sample) or EMM with 5 mM EDTA for 8 h (+EDTA sample). Ecl1‐GST protein was purified using COSMOGEL GST‐Accept (09277, Nacalai Tesque Inc.).

### 4.6 Mass spectrometry analysis of phosphorylated amino acid residues

Purified GST‐tagged protein samples were dissolved in a sample buffer and separated using sodium dodecyl sulfate‐polyacrylamide gel electrophoresis. In‐gel digestion was performed using trypsin/Lys‐C mix, chymotrypsin/trypsin/Lys‐C mix, chymotrypsin, or Lys‐C (Promega) with ProteaseMAX Surfactant (Promega). The digested peptides were analyzed by nanoflow reverse‐phase LC followed by tandem MS using a Q‐Exactive hybrid mass spectrometer (Thermo Fisher Scientific). The desalted peptides were loaded into a separation capillary C18 reverse‐phase column (NTCC‐360/100–3–125, 125 × 0.1 mm^2^; Nikkyo Technos). Xcalibur v3.0.63 software (Thermo Fisher Scientific) was used to record the peptide spectra over the mass range of m/z 350–1800. MS spectra were repeatedly recorded followed by 10 data‐dependent high energy collisional dissociation MS/MS spectra generated from the 10 highest intensity precursor ions. These spectra were interpreted, and peak lists were generated using Proteome Discoverer software v2.2.0.388 and v2.4.1.15 (Thermo Fisher Scientific). The SEQUEST algorithm in Proteome Discoverer was used to perform a search against the Ecl1‐GST sequence, and the Ecl1‐GST sequence was also searched for contaminants using the cRAP database (https://www.thegpm.org/crap/). The search parameters were as follows: enzyme selected with two (trypsin, Lys‐C) or four (chymotrypsin) maximum missing cleavage sites, mass tolerance of 10 ppm for peptide tolerance, 0.02 Da for MS/MS tolerance, fixed modification of carbamidomethyl (C), and variable modification of oxidation (M) and phosphorylation (S, T, Y). Peptide identifications were based on significant Xcorr values (high confidence filter). Modified peptides identified by Proteome Discoverer were normalized by the target protein sequence and quantified using label‐free quantification.

### 4.7 Measurement of mating rate

All data were calculated by counting at least 300 cells. Homothallic haploid cells (*h* ^90^) were cultured in EMM for 3 days at 30°C. The mating frequency (*Fm*) was calculated as a percentage according to the equation *Fm* = (2*Z* + 2*A* + 0.5*S*)/(*H* + 2*Z* + 2*A* + 0.5*S*), where *H* was the number of unmated haploid cells, *Z* was the number of zygotes, *A* was the number of asci, and *S* was the number of free spores.

### 4.8 CLS analyses

Cells were cultured in synthetic SD medium without thiamine (Ohtsuka et al., 2024a) to determine the survival rate of those harboring pnmt1‐Ecl1‐GST during the stationary phase. To measure CLS, using colony‐forming units, the numbers of viable cells in 1 mL aliquots of culture were determined and divided by cell turbidity at each sampling time. To determine cell survival under sulfur starvation, cells were cultured in SD medium to OD_600 nm_ = 0.5 and then transferred into sulfur‐free SD medium (Shimasaki et al., 2020) for 3 days. Cell growth was measured by monitored by turbidity using a BACT‐550 Bactomonitor equipped with a 600 nm filter (Nissho Denki Co).

### 4.9 Western blot analysis

Western blot was performed as previously described (Hibi et al., 2018). Ecl1‐GFP and GFP were detected using anti‐GFP (11,814,460, Roche), Ecl1‐GST and GST using monoclonal anti‐GST (G1160, Sigma), Mip1‐HA using anti‐HA (12CA5) (11,583,816, Roche), phosphorylated Psk1 using phospho‐p70 S6 kinase (Thr389) (1A5) mouse mAb (9206, Cell Signaling Technology), Psk1‐myc using MYC/c‐Myc antibody (sc‐40, Santa Cruz Biotechnology), α‐tubulin using monoclonal anti‐α‐tubulin antibody (T6074, Sigma‐Aldrich), and γ‐tubulin using the monoclonal anti‐Gtb1/γ‐tubulin antibody (T5326, Sigma‐Aldrich).

### 4.10 Pull‐down assay for investigation of Ecl1 and Mip1 interaction

Cells in lysis buffer (50 mM Tris [pH 8.0], 150 mM NaCl, 5 mM EDTA, 0.1% NP40, and 1× Roche complete protease inhibitor cocktail without EDTA) were lysed using an MB3200(S) Multibeads Shocker (Yasui Kikai). Ecl1‐GST protein was purified using COSMOGEL GST‐Accept. Mip1‐HA protein was purified using anti‐HA antibody beads (014–23,081, Fujifilm).

### 4.11 Flow cytometry analysis

Flow cytometry analysis was performed using an Attune Acoustic Focusing Cytometer (Life Technologies) to measure the DNA content. Briefly, the cells were fixed with 70% ethanol and treated with an RNase solution (R6148, Sigma) for 90 min at 37°C. The nucleic acids were then stained with propidium iodide (PI) before flow cytometry analysis. Over 10,000 cells were examined for each sample. The PI intensity was detected at a voltage of 2.55 V on the BL3 channel.

### 4.12 Calculation of doubling time

The doubling time (*D*) was calculated according to the equation *D* = ln 2 /*μ* and *μ* = (ln *X* − ln *X*_*0*_)/(*t* − *t*_*0*_), where *X* and *X*_*0*_ are the turbidity at time *t* or *t*_*0*_, respectively.

### 4.13 Statistical analysis

Quantitative data presented in the figures represent the average of at least three independent experiments ± standard deviation. Statistical analyses were performed by two‐tailed unpaired Student *t* tests.

### 4.14 AlphaFold prediction and modeling

Protein structure was predicted using AlphaFold 2 modeling with AlphaFold Colab prediction tools (https://colab.research.google.com/github/sokrypton/ColabFold/blob/main/AlphaFold2.ipynb) (Mirdita et al., 2022), and the Protein Data Bank files were downloaded.

## AUTHOR CONTRIBUTIONS

*Conceptualization*: HO, HA; *Investigation*: SK, YK, YI, TS, KI, YO, EM, KK; *Writing – original draft*: HO; *Writing – review & editing*: YO, AY, HA; Supervision: HA; Project administration: HA; *Funding acquisition*: HO, HA.

## CONFLICT OF INTEREST STATEMENT

The authors state they have no competing interests or disclosures.

## Supporting information


Table S1.

Figure S1.
**Figure S2**.
**Figure S3**.

## Data Availability

All data generated or analyzed during this study can be found within the article and its supplemental information.
