# Supplementary material for: Novel TORC1 inhibitor Ecl1 is regulated by phosphorylation in fission yeast
Source: Aging Cell. 2025 Feb 5;24(4):e14450. doi: 10.1111/acel.14450 (PMC11984688; doi:10.1111/acel.14450)
Supplement: Supplementary file 2 — Figure S1. Figure S2. Figure S3. [file ACEL-24-e14450-s001.pdf]

Ecl1-T7-Phospho

Peptide Summary

Sequence: MDLDFCTVCGATTQDGSLYCSSECHLLDFTK, C6-Carbamidomethyl (57.02146 Da), C9-Carbamidomethyl (57.02146 Da), C20-Carbamidomethyl (57.02146 Da), C24-Carbamidomethyl (57.02146 Da), T7-Phospho (79.96633 Da)
Charge: +4, Monoisotopic m/z: 928.61993 Da (-5.97 mmu/-6.43 ppm), MH+: 3711.45787 Da, RT: 31.9388 min,
Identified with: Sequest HT (v1.17); XCorr:4.91, ptmRS: Best Site Probabilities:T7(Phospho): 98.02,
Fragment match tolerance used for search: 0.02 Da

Fragment Matches

Value Type: Theo. Mass [Da]

Ion Series | Modification Losses | Neutral Losses | Multiple Neutral Losses | Precursor Ions | Internal Fragments

#1	b^+	b^{2+}	b^{3+}	b^{4+}	Seq.	y^+	y^{2+}	y^{3+}	y^{4+}	#2
1	132.04776	66.52752	44.68744	33.76740	M					31
2	247.07470	124.04099	83.02975	62.52413	D	3580.44125	1790.72426	1194.15193	895.86577	30
3	360.15877	180.58302	120.72444	90.79515	L	3465.41431	1733.21079	1155.80962	867.10903	29
4	475.18571	238.09649	159.06675	119.55189	D	3352.33024	1676.66876	1118.11493	838.83802	28
5	622.25412	311.63070	208.08956	156.31899	F	3237.30330	1619.15529	1079.77262	810.08128	27
6	782.28477	391.64602	261.43311	196.32665	C-Carbami...	3090.23489	1545.62108	1030.74981	773.31418	26
7	963.29878	482.15303	321.77111	241.58015	T-Phospho	2930.20424	1465.60576	977.40626	733.30652	25
8	1062.36720	531.68724	354.79392	266.34726	V	2749.19023	1375.09875	917.06826	688.05301	24
9	1222.39785	611.70256	408.13747	306.35492	C-Carbami...	2650.12181	1325.56454	884.04546	663.28591	23
10	1279.41931	640.21329	427.14462	320.61028	G	2490.09116	1245.54922	830.70191	623.27825	22
11	1350.45642	675.73185	450.82366	338.36956	A	2433.06970	1217.03849	811.69475	609.02288	21
12	1451.50410	726.25569	484.50622	363.63148	T	2362.03259	1181.51993	788.01571	591.26360	20
13	1552.55178	776.77953	518.18878	388.89340	T	2260.98491	1130.99609	754.33315	566.00168	19
14	1680.61036	840.80882	560.87497	420.90805	Q	2159.93723	1080.47225	720.65059	540.73977	18
15	1795.63730	898.32229	599.21728	449.66478	D	2031.87865	1016.44296	677.96440	508.72512	17
16	1852.65876	926.83302	618.22444	463.92015	G	1916.85171	958.92949	639.62209	479.96839	16
17	1939.69079	970.34903	647.23512	485.67816	S	1859.83025	930.41876	620.61493	465.71302	15
18	2052.77486	1026.89107	684.92980	513.94917	L	1772.79822	886.90275	591.60426	443.95501	14
19	2215.83818	1108.42273	739.28425	554.71500	Y	1659.71415	830.36072	553.90957	415.68400	13
20	2375.86883	1188.43805	792.62780	594.72267	C-Carbami...	1496.65083	748.82905	499.55513	374.91816	12
21	2462.90086	1231.95407	821.63847	616.48067	S	1336.62018	668.81373	446.21158	334.91050	11
22	2549.93289	1275.47008	850.64915	638.23868	S	1249.58815	625.29771	417.20090	313.15249	10
23	2678.97548	1339.99138	893.66335	670.49933	E	1162.55612	581.78170	388.19022	291.39449	9
24	2839.00613	1420.00670	947.00689	710.50699	C-Carbami...	1033.51353	517.26040	345.17603	259.13384	8
25	2976.06504	1488.53616	992.69320	744.77172	H	873.48288	437.24508	291.83248	219.12618	7

Fragment Spectrum

20180105_QE1_sand_AcOH_PE_2018-002_e1-1_02.raw #14075 RT: 31.9388 min
FTMS, 929.1270@hcd30.00, z=+4, Mono m/z=928.61993 Da, MH+=3711.45787 Da, Match Tol.=0.02 Da

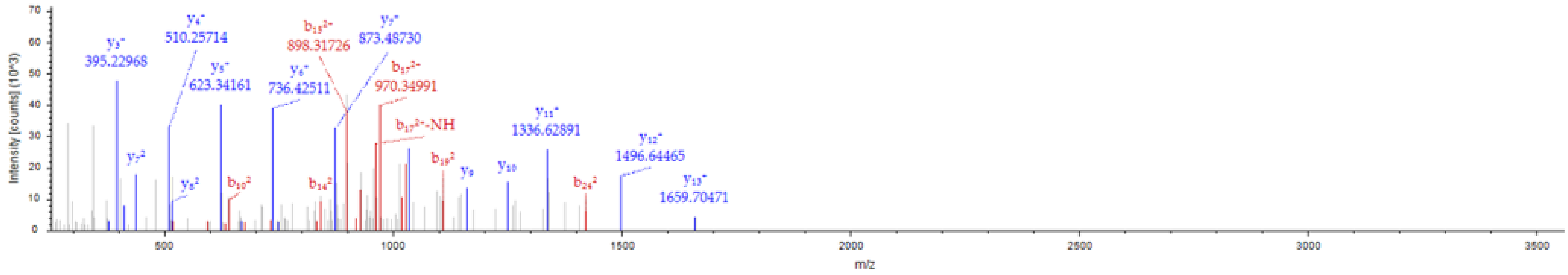

Ecl1-S22-Phospho

Peptide Summary

Sequence: CSSECHLLDFTK, C1-Carbamidomethyl (57.02146 Da), C5-Carbamidomethyl (57.02146 Da), S3-Phospho (79.96633 Da)
Charge: +2, Monoisotopic m/z: 788.81186 Da (-0.36 mmu/-0.45 ppm), MH+: 1576.61645 Da, RT: 16.4831 min,
Identified with: Sequest HT (v1.17); XCorr:3.45, ptmRS: Best Site Probabilities:S3(Phospho): 100,
Fragment match tolerance used for search: 0.02 Da

Fragment Matches

Value Type: Theo. Mass [Da]

Ion Series | Modification Losses | Neutral Losses | Multiple Neutral Losses | Precursor Ions | Internal Fragments

#1	b^+	b^{2+}	Seq.	y^+	y^{2+}	#2
1	161.03793	81.02260	C-Carbami...			12
2	248.06995	124.53862	S	1416.58651	708.79689	11
3	415.06831	208.03779	S-Phospho	1329.55448	665.28088	10
4	544.11091	272.55909	E	1162.55612	581.78170	9
5	704.14155	352.57442	C-Carbami...	1033.51353	517.26040	8
6	841.20047	421.10387	H	873.48288	437.24508	7
7	954.28453	477.64590	L	736.42397	368.71562	6
8	1067.36859	534.18794	L	623.33990	312.17359	5
9	1182.39554	591.70141	D	510.25584	255.63156	4
10	1329.46395	665.23561	F	395.22890	198.11809	3
11	1430.51163	715.75945	T	248.16048	124.58388	2
12			K	147.11280	74.06004	1

Fragment Spectrum

20180105_QE1_sand_AcOH_PE_2018-002_e3-2_01.raw #7364 RT: 16.4831 min
FTMS, 788.8137@hcd30.00, z=+2, Mono m/z=788.81186 Da, MH+=1576.61645 Da, Match Tol.=0.02 Da

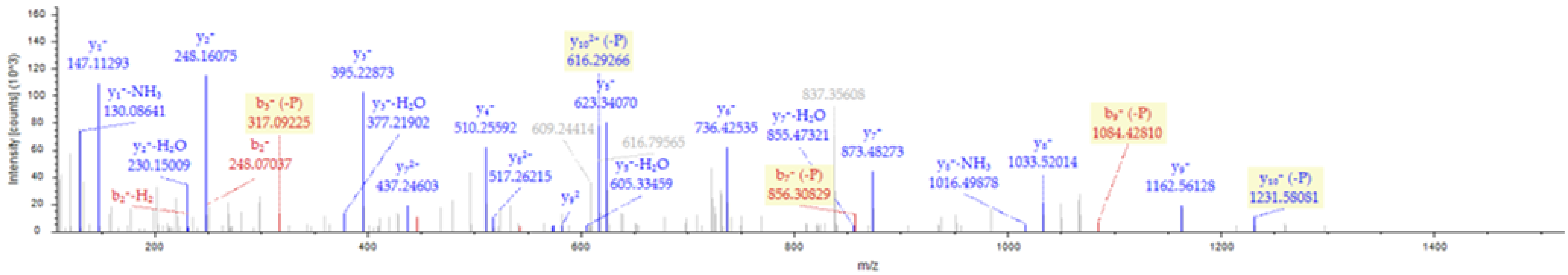

Ohtsuka et al. Fig. S1

Ecl1-S61-Phospho

Peptide Summary

Sequence: HRKSMTSADFPTPRFSAY, S4-Phospho (79.96633 Da), M5-Oxidation (15.99492 Da)
Charge: +3, Monoisotopic m/z: 732.33054 Da (+1.04 mmu/+1.41 ppm), MH+: 2194.97707 Da, RT: 14.6048 min,
Identified with: Sequest HT (v1.17); XCorr:4.38, ptmRS: Best Site Probabilities:S4(Phospho): 99.22; M5(Oxidation): 100,
Fragment match tolerance used for search: 0.02 Da

Fragment Matches

Value Type: Theo. Mass [Da]

Ion Series | Modification Losses | Neutral Losses | Multiple Neutral Losses | Precursor Ions | Internal Fragments

#1	b+	b2+	b3+	Seq.	y+	y2+	y3+	#2
1	138.06619	69.53673	46.69358	H				18
2	294.16730	147.58729	98.72728	R	2057.91505	1029.46116	686.64320	17
3	422.26226	211.63477	141.42561	K	1901.81394	951.41061	634.60950	16
4	589.26062	295.13395	197.09172	S-Phospho	1773.71898	887.36313	591.91118	15
5	736.29602	368.65165	246.10352	M-Oxidation	1606.72062	803.86395	536.24506	14
6	837.34370	419.17549	279.78608	T	1459.68522	730.34625	487.23326	13
7	924.37573	462.69150	308.79676	S	1358.63754	679.82241	453.55070	12
8	995.41284	498.21006	332.47580	A	1271.60551	636.30639	424.54002	11
9	1110.43978	555.72353	370.81811	D	1200.56840	600.78784	400.86098	10
10	1257.50820	629.25774	419.84092	F	1085.54146	543.27437	362.51867	9
11	1354.56096	677.78412	452.19184	P	938.47304	469.74016	313.49587	8
12	1455.60864	728.30796	485.87440	T	841.42028	421.21378	281.14494	7
13	1552.66140	776.83434	518.22532	P	740.37260	370.68994	247.46238	6
14	1708.76252	854.88490	570.25902	R	643.31984	322.16356	215.11146	5
15	1855.83093	928.41910	619.28183	F	487.21873	244.11300	163.07776	4
16	1942.86296	971.93512	648.29250	S	340.15031	170.57879	114.05496	3
17	2013.90007	1007.45367	671.97154	A	253.11828	127.06278	85.04428	2
18				Y	182.08117	91.54422	61.36524	1

Fragment Spectrum

20180105_QE1_sand_AcOH_PE_2018-002_e3-3_01.raw #6120 RT: 14.6048 min
FTMS, 732.6660@hcd30.00, z=+3, Mono m/z=732.33054 Da, MH+=2194.97707 Da, Match Tol.=0.02 Da

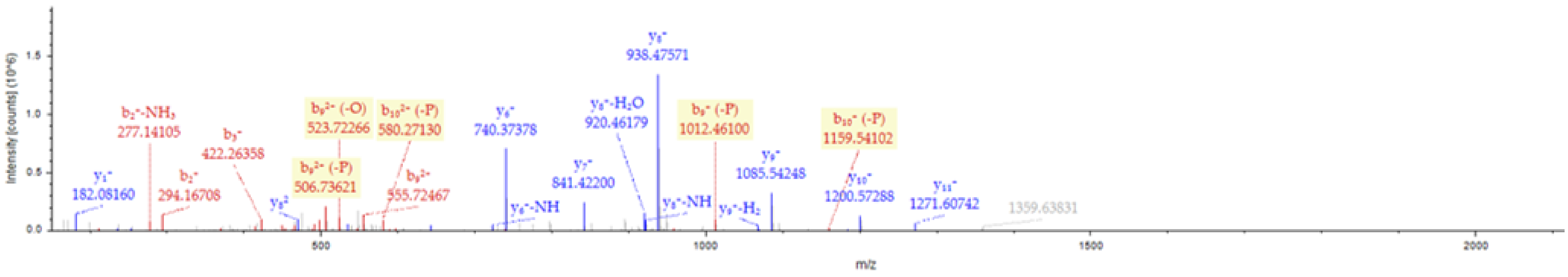

Ecl1-S61, T63, T69-Phospho

Peptide Summary

Sequence: SMTSADFPTPRFSAYTK, T3-Phospho (79.96633 Da), T9-Phospho (79.96633 Da)
Charge: +3, Monoisotopic m/z: 689.61468 Da (-1.18 mmu/-1.72 ppm), MH+: 2066.82949 Da, RT: 18.1072 min,
Identified with: Sequest HT (v1.17); XCorr:4.19, ptmRS: Best Site Probabilities:S1(Phospho): 99.99; T9(Phospho): 100,
Fragment match tolerance used for search: 0.02 Da

Fragment Matches

Value Type: Theo. Mass [Da]

Ion Series | Modification Losses | Neutral Losses | Multiple Neutral Losses | Precursor Ions | Internal Fragments

#1	b+	b2+	b3+	Seq.	y+	y2+	y3+	#2
1	88.03930	44.52329	30.01795	S				17
2	219.07979	110.04353	73.69811	M	1979.80101	990.40414	660.60519	16
3	400.09380	200.55054	134.03612	T-Phospho	1848.76052	924.88390	616.92503	15
4	487.12583	244.06655	163.04679	S	1667.74651	834.37690	556.58702	14
5	558.16294	279.58511	186.72583	A	1580.71449	790.86088	527.57635	13
6	673.18988	337.09858	225.06815	D	1509.67737	755.34232	503.89731	12
7	820.25830	410.63279	274.09095	F	1394.65043	697.82885	465.55499	11
8	917.31106	459.15917	306.44187	P	1247.58202	624.29465	416.53219	10
9	1098.32507	549.66617	366.77987	T-Phospho	1150.52925	575.76826	384.18127	9
10	1195.37783	598.19256	399.13080	P	969.51524	485.26126	323.84327	8
11	1351.47895	676.24311	451.16450	R	872.46248	436.73488	291.49234	7
12	1498.54736	749.77732	500.18730	F	716.36137	358.68432	239.45864	6
13	1585.57939	793.29333	529.19798	S	569.29295	285.15011	190.43584	5
14	1656.61650	828.81189	552.87702	A	482.26092	241.63410	161.42516	4
15	1819.67983	910.34355	607.23146	Y	411.22381	206.11554	137.74612	3
16	1920.72751	960.86739	640.91402	T	248.16048	124.58388	83.39168	2
17				K	147.11280	74.06004	49.70912	1

Fragment Spectrum

20180105_QE1_sand_AcOH_PE_2018-002_e1-4_01.raw #7407 RT: 18.1072 min
FTMS, 689.9534@hcd30.00, z=+3, Mono m/z=689.61468 Da, MH+=2066.82949 Da, Match Tol.=0.02 Da

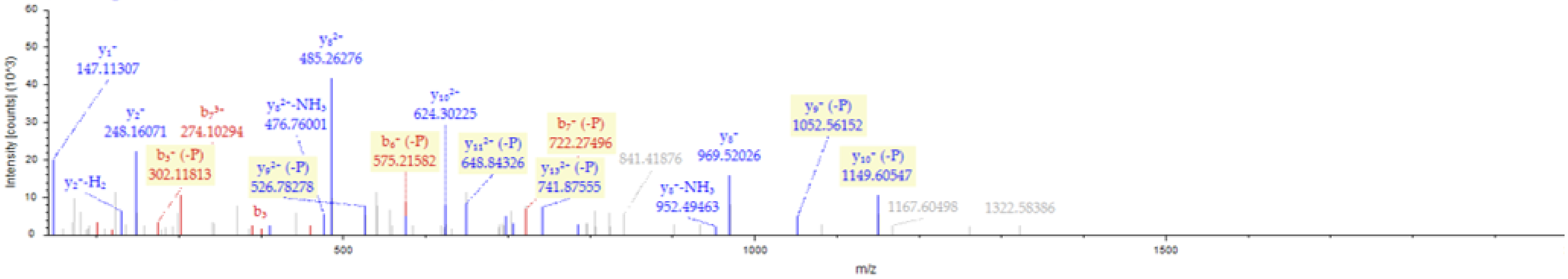

Ohtsuka et al. Fig. S1

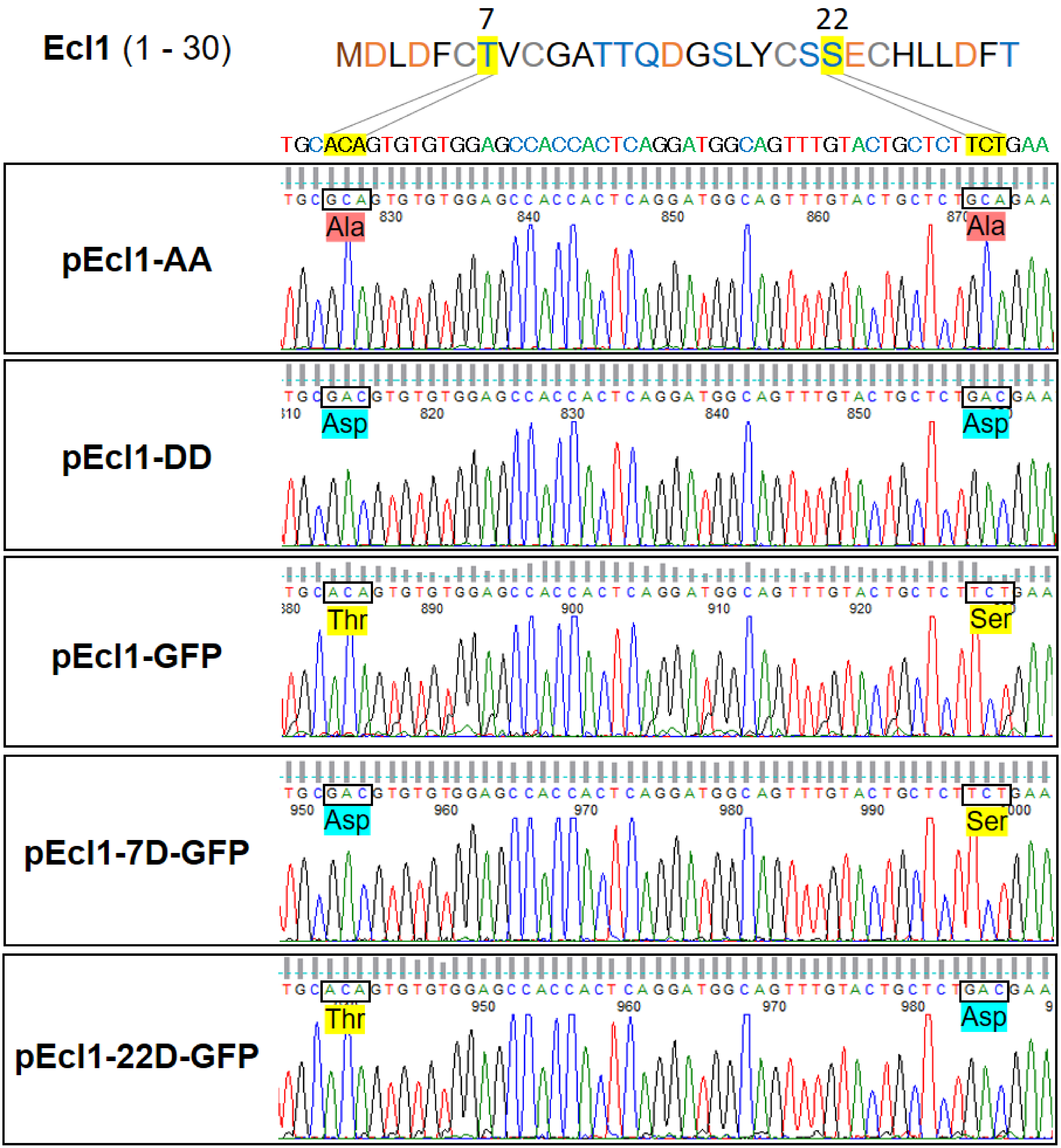

Ohtsuka et al. Fig. S2

Serine type

```
Sp Ecl1          18 LYCSSECHL 26
Km KLMA_10593    29 LYCSSQCQE 37
```

Positively charged type

```
Sp Ecl2          17 LYCSRECHL 25
Sj Ecl1          17 LYCSRECHL 25
```

Polar uncharged type

```
Sp Ecl3          18 LYCSNECRI 26
```

Hydrophobic type

```
Rt RHTO_02967    38 PYCSAECRK 46
```

Negatively charged type

```
Sc Ecl1          45 LYCSEDCKL 53
Mr COH1          24 AYCSESCRL 32
Lr LRAMOSA01014  21 LYCSEECLR 29
Ma 0023d01954    22 LYCSEDCLR 30
Ma 0041c02951    21 LYCSDQCLK 29
Ng CAGL0E03025g  20 IYCSEECKH 28
Pm PICMED_100613 20 AYCSDTCKL 28
Rm BCV71D_83866  21 LYCSEQCLR 29
```

Ohtsuka et al. Fig. S3
